# Prolactin and endocrine therapy resistance in breast cancer: The next potential hope for breast cancer treatment

**DOI:** 10.1111/jcmm.16946

**Published:** 2021-10-15

**Authors:** Yuan Li, Xiangyi Kong, Lixue Xuan, Zhongzhao Wang, Yen‐Hua Huang

**Affiliations:** ^1^ Department of Breast Surgical Oncology National Cancer Center/National Clinical Research Center for Cancer/Cancer Hospital Chinese Academy of Medical Sciences and Peking Union Medical College Beijing China; ^2^ Department of Biochemistry and Molecular Cell Biology School of Medicine College of Medicine Taipei Medical University Taipei Taiwan

**Keywords:** breast cancer, endocrine therapy, prolactin, prolactin receptor, resistance, review

## Abstract

Breast cancer, a hormone‐dependent tumour, generally includes four molecular subtypes (luminal A, luminal B, HER2 enriched and triple‐negative) based on oestrogen receptor, progesterone receptor and human epidermal growth factor receptor‐2. Multiple hormones in the body regulate the development of breast cancer. Endocrine therapy is one of the primary treatments for hormone‐receptor‐positive breast cancer, but endocrine resistance is the primary clinical cause of treatment failure. Prolactin (PRL) is a protein hormone secreted by the pituitary gland, mainly promoting mammary gland growth, stimulating and maintaining lactation. Previous studies suggest that high PRL levels can increase the risk of invasive breast cancer in women. The expression levels of PRL and PRLR in breast cancer cells and breast cancer tissues are elevated in most ER^+^ and ER^−^ tumours. PRL activates downstream signalling pathways and affects endocrine therapy resistance by combining with prolactin receptor (PRLR). In this review, we illustrated and summarized the correlations between endocrine therapy resistance in breast cancer and PRL, as well as the pathophysiological mechanisms and clinical practices. The study on PRL and its receptor would help explore reversing endocrine therapy‐resistance for breast cancer.

## 1 INTRODUCTION

The incidence of breast cancer ranks first among Chinese female malignant tumours. Even in women worldwide, breast cancer is also the malignant tumour with the highest incidence and mortality and accounts for about 11.6% of total cancer deaths.^1^ In addition to operation and chemotherapy, endocrine therapy is also a common therapy for breast cancer. According to studies, endocrine therapy is effective in 50%–60% of patients with positive oestrogen receptor (ER); the response rate (RR) of endocrine therapy in patients with positive ER and progesterone receptor (PR) may be >75% and that in patients with negative ER and PR is about 10%.^2^ Endocrine therapies for ER^+^ breast cancer include selective ER modulators (SERMs) such as tamoxifen, aromatase inhibitors (AIs) such as anastrozole, ovarian function suspension (OFS) such as goserelin and selective ER downregulators (SERDs) such as fulvestrant. The main reason for endocrine therapy's failure is the primary or secondary resistance in patients during treatment. Primary resistance is common in breast cancer patients with negative ER and PR. Nevertheless, a considerable part of patients with positive ER also has primary resistance. However, after a certain period of endocrine therapy, almost all patients may have secondary resistance. Therefore, endocrine resistance is a crucial problem to be solved in the treatment of breast cancer.

Prolactin's (PRL) role in breast cancer pathogenesis has been gaining increasing attention. PRL is a protein hormone primarily secreted by eosinophils in the anterior lobe of the pituitary gland. PRL can stimulate DNA synthesis, epithelial cell proliferation and breast milk production by affecting the cell of origin or neighbouring cells in an autocrine/paracrine way and prolactin receptors.^3^ PRLR, a member of the cytokine receptor family, mediates the growth regulation of PRL on the human breast. In breast cancer studies, these data demonstrated widespread expression of PRL and its receptor (>95%).^4^

An epidemiological survey has proved that the elevated PRL level in the circulation before the confirmation of breast cancer is correlated to metastatic breast cancer (MBC).^5^ Breast cells can promote cell proliferation and inhibit cell apoptosis through autocrine or paracrine PRL. PRLR is found in breast tissue. The expression levels of PRL and PRLR in breast cancer cells and breast cancer tissues are elevated in most ER^+^ and ER^−^ tumours.^6^ The study of He Wei et al. found that PRL can boost the proliferation and growth of human breast cancer T‐47D cells and accelerate the transformation of the cell growth cycle and that the effect is dose‐ and time‐dependent.^7^ In the Nurse's Health Study and Nurse's Health Study II, Tvoroger et al. investigated the relationship between PRL and breast cancer risk. They measured PRL levels <10 and ≥10 years before the diagnosis of breast cancer. After 20 years of follow‐up, the finding has demonstrated an association of prolactin levels <10 years before diagnosis and breast cancer risk of postmenopausal women, especially for ER(+) tumours and metastatic disease.^8^ In another case control study, Tikk et al. analysed the association of PRL prediagnostic circulatory levels with the risk of breast cancer through menopause status, HRT therapy with the use of a blood donation system and the hormone receptor status of the breast tumours in 2250 breast cancer cases, the study found that the risk of breast cancer among postmenopausal women was higher because of prolactin overexpression. However, this increased risk was limited to women who used postmenopausal HRT when donating blood.^9^ No evidence existed in other studies that combined hormone therapy increased serum PRL levels in hormone replacement therapy patients.^10^, ^11^

In addition, knocking down the long PRLR gene can inhibit breast cancer's metastasis in the lung and liver, indicating that the long PRLR gene plays a crucial role in breast cancer metastasis.^12^ Researchers have demonstrated in Yonezawa's study that the long PRLR associate with breast cancer metastasis by knocking down the long PRLR in two breast cancer models. In both models, knockdown of long PRLR dramatically inhibited lungs and liver metastasis.^12^ Sutherland et al. used quantitative immunohistochemistry to identify the associations between PRLR levels and time to bone metastasis, and they observed that PRL‐PRLR can accelerate bone metastasis in breast cancer and the PRLR overexpression in the primary breast tumour results in a shorter time to metastasis.^13^ Another study revealed no differences in serum PRL levels among visceral or bone metastases, so further studies are necessary to examine the association of PRL with breast cancer metastasis.^14^ All these data indicated that PRL is closely correlated to the tumourigenesis and development of breast cancer.

Hachim et al. used tissue microarray (TMA) in 102 patients to analyse the relationship of PRLR expression levels and different breast cancer molecular subtypes. They found the PRLR was present in luminal A (30%), luminal B (28.5%) and Her2‐enriched (23.08%), but not in triple‐negative patients.^15^ They conducted a larger analysis in GOBO database (1881 patients) to assess PRLR gene expression level and proved that there was a significant association between PRLR expression and the luminal A subtype, but they also found no significant link between PRLR expression and hormone receptors. In the future, the relevance of PRL and molecular subtypes of breast cancer requires further study.

As is known, the independent activation of ER ligand is one of the mechanisms of endocrine therapy resistance. The evidence suggested that PRL may cause endocrine resistance through this mechanism in vitro.^16^ The study of O'Leary et al.^17^ showed that PRL may also activate ER in the absence of oestrogen ligand in vivo, causing endocrine resistance. In this study, the correlation between PRL and breast cancer endocrine resistance was reviewed.

## 2 ENDOCRINE RESISTANCE MECHANISMS

**FIGURE 1:**
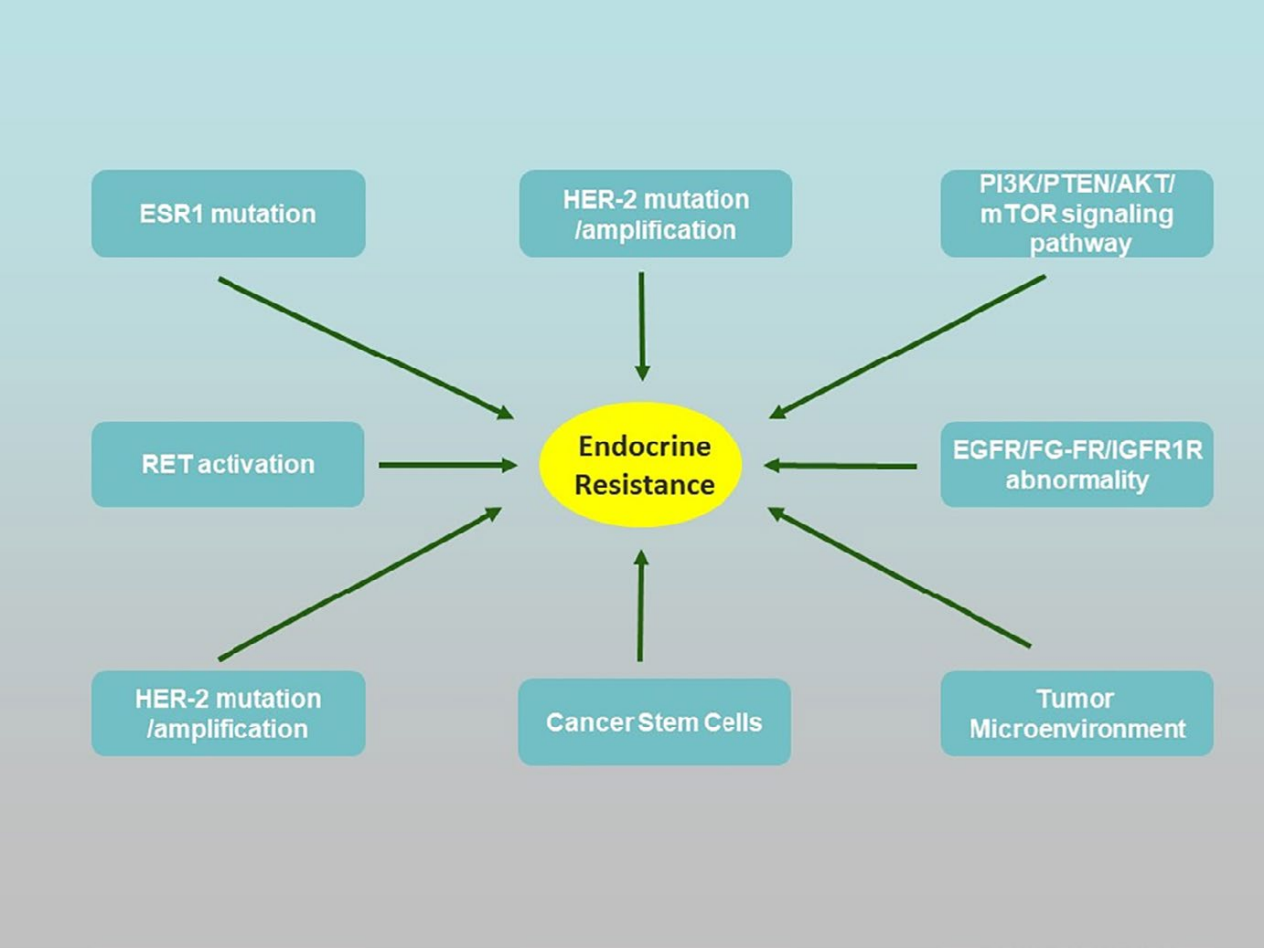
Mechanisms of endocrine resistance. ESR1, oestrogen receptor 1; HER‐2, human epidermal growth factor receptor 2; RET, rearranged during transfection

Endocrine resistance is mainly divided into primary resistance and secondary resistance. The mechanisms of primary resistance mainly include: induction of apparent silence of ER by histone deacetylation modification, ER gene mutation (such as conversion of *ERα351* site tyrosine to aspartic acid), promotion of tamoxifen (TAM) to act as an agonist and tumour growth, gene mutation of ER alpha (*ESR*) and p21‐activated kinase 1 *(Pak* *1*) and aromatase gene polymorphism.^17^ *ESR1* and *Pak1* gene mutation play a vital role in the endocrine therapy resistance of breast cancer with positive ER.^18^, ^19^ Secondary resistance is correlated to the expression and functional down‐regulation of ERα, the overexpression of ERβ of breast cancer with positive ERα, ligand‐independent activation of growth factor receptor (including epidermal growth factor receptor [EGFR], human epidermal growth factor receptor‐2 [HER‐2] and insulin‐like growth factor 1 receptor [IGF1‐R]) or intracellular kinases (such as mitogen‐activated protein kinase [MAPK]), and gene transcription caused by ER phosphorylation due to the activation of the downstream signal transduction pathway from protein kinase A (PKA) phosphorylation. Besides, the positive gene of fibroblast growth factor receptor 1 (*FGFR1*) is also correlated to breast cancer resistance.^20^, ^21^, ^22^ In the mechanism of endocrine resistance, ER missing accounts for 15%–20% and ER mutation <1%.^23^ Figure 1 briefly depicts the mechanism of endocrine resistance.

### 2.1 Receptor‐mediated signalling in endocrine therapy resistance

#### 2.1.1 Oestrogen receptor‐mediated signalling

**FIGURE 2:**
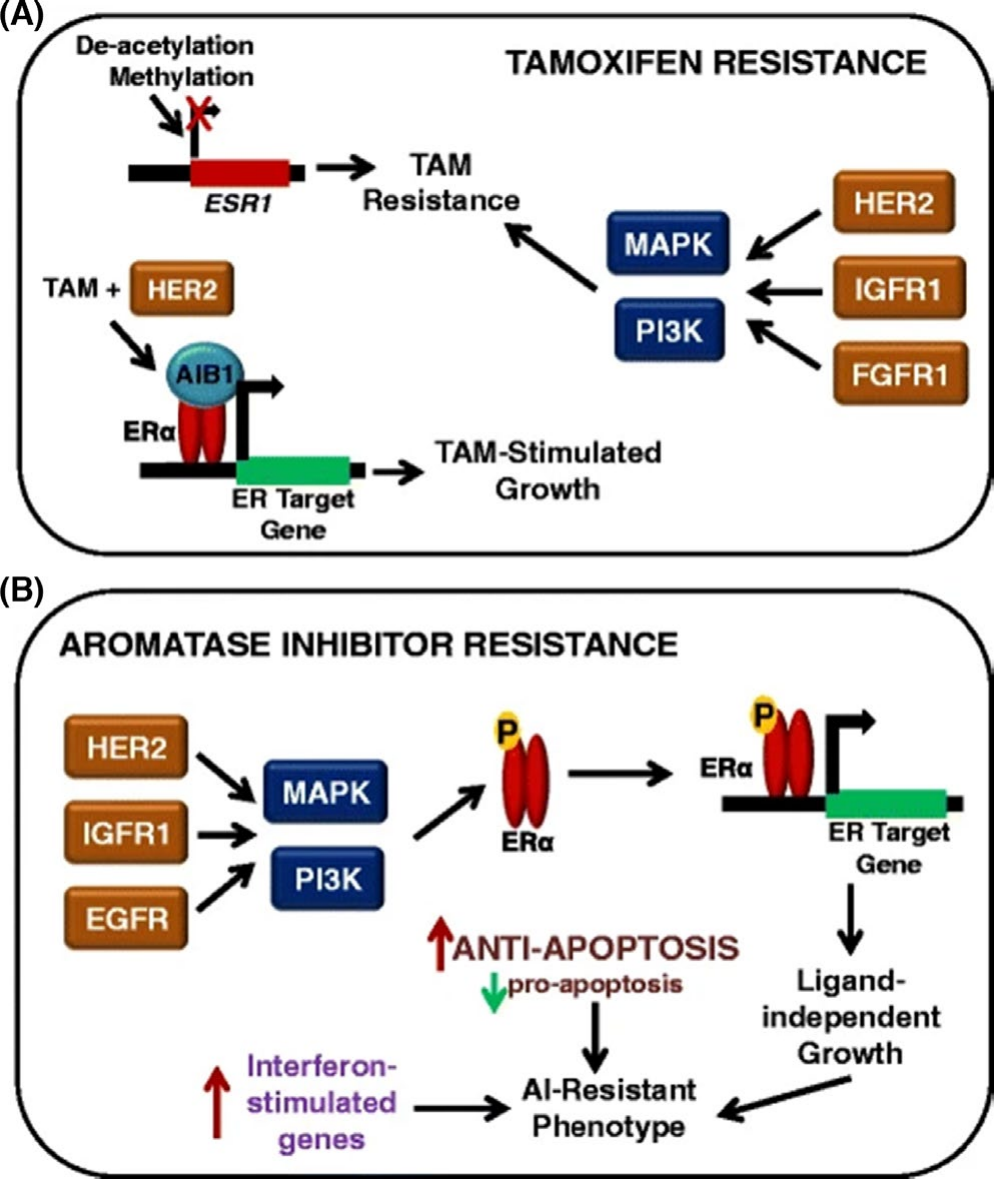
Mechanisms of endocrine resistance in breast cancer cells. (A) Mechanisms of tamoxifen (TAM) resistance may involve the loss of oestrogen receptor (ER) alpha expression, which can be achieved by methylation of CpG islands or histone deacetylase activity in the ESR1 promoter. Tamoxifen‐resistant growth is also stimulated by the upregulation of growth factor signalling pathways (HER2, IGF‐IR and FGFR) and subsequent activation of the mitogen‐activated protein kinase (MAPK) cascade or phosphoinositide 3‐kinase (PI3K) pathway. Finally, tamoxifen has even been shown to stimulate the growth of breast cancer cells when bound to certain coactivators, such as AIB1, and this is especially true in HER2‐expressing cells. (B) The mechanisms of aromatase inhibitor (AI) resistance share similarities with tamoxifen resistance, especially in terms of growth factor pathway upregulation. The enhanced activity of growth factors such as MAPK can result in oestrogen‐independent phosphorylation and activation of ERα. In addition to growth factor signalling, interferon response genes and anti‐apoptotic proteins have also been shown to have increased expression in AI‐resistant cells. AIB1, amplified in breast cancer 1; FGFR1, fibroblast growth factor receptor 1; HER2, human epidermal growth factor receptor 2; IGFR1, insulin‐like growth factor receptor 1. Reprinted from [24]. Copyright © 2015 Breast Cancer Research volume

The interaction between ER signalling and other receptor‐mediated signalling plays a crucial part in the therapeutic process for endocrine resistance. Blocking the oestrogen‐mediated signalling and other over‐activated signalling may reverse the resistance of endocrine therapy. The combination of Tamoxifen and growth factor receptor kinase inhibitor (RKI) is also one of the main therapeutic approaches for Tamoxifen‐resistant breast cancer with overexpression of EGFR or HER‐2. In addition, the abnormal expression of receptor tyrosine kinase (RTK), EGFR, HER‐2, IGF‐1R, fibroblast growth factor receptors (FGF‐R) and abnormal activation such as PI3K/PTEN/AKT/mTOR signalling pathway, NF‐kB signalling pathway may be involved in drug resistance (Figure 2).^24^

#### 2.1.2 HER‐2

**FIGURE 3:**
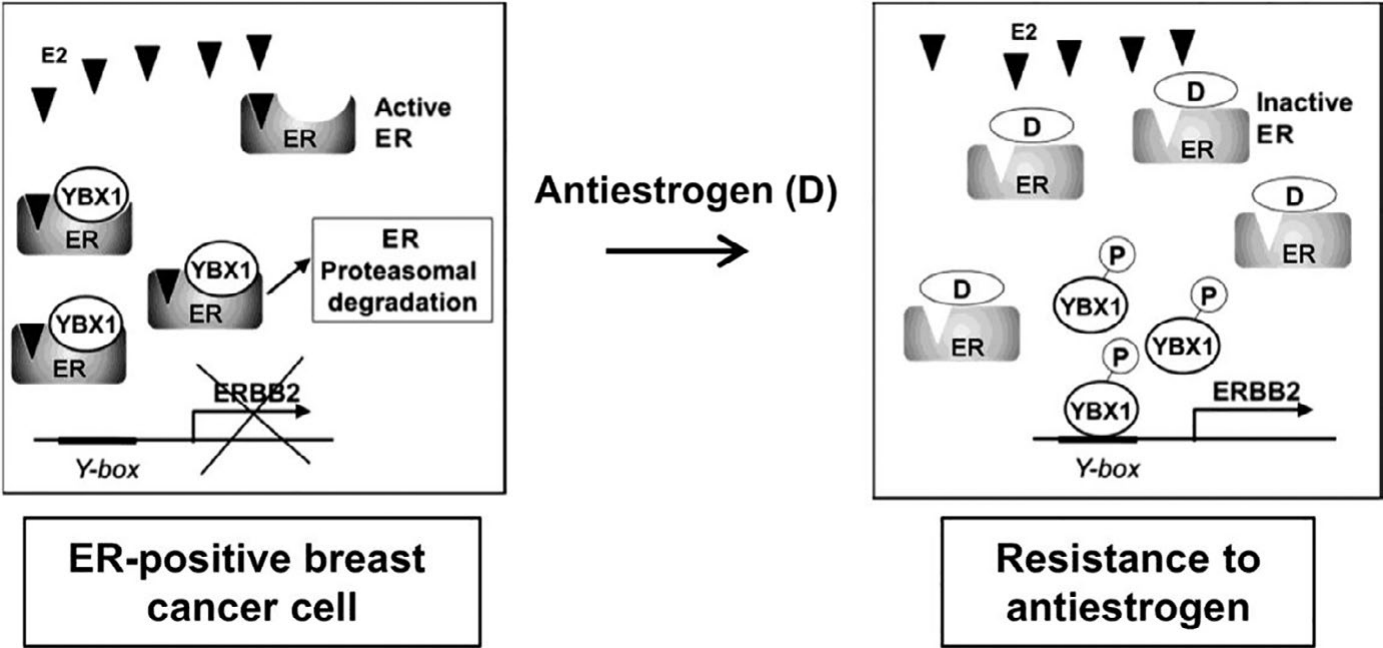
Model depicting YBX1‐mediated resistance to anti‐oestrogens of breast cancer cells. In oestrogen‐dependent ER^+^ breast cancer cells, YBX1‐induced ERBB2 expression is inhibited by YBX1 binding to active ER. Treatment with anti‐oestrogens interferes with binding, and free, active YBX1 promotes ERBB2 expression. Reprinted from [27]. Copyright © 2017 Cancer Res

HER‐2 is involved in resistance to endocrine therapy. Studies have found that tamoxifen‐resistant cells overexpress HER‐2. After tamoxifen treatment, the cells still proliferate malignantly, suggesting that HER‐2 interacts with ERα.^25^ Another study found that breast cancer amplified antigen 1 (AIB1) as an ERα co‐regulator when HER‐2 is expressed, and its increased expression is related to tamoxifen resistance.^26^ YBX1 overexpressing breast cancer cells are resistant to tamoxifen and fulvestrant, which are related to decreased ER and elevated HER‐2. Tamoxifen treatment can increase the ability of YBX1 to bind to the HER‐2 promoter region, Induction of HER‐2 transcriptional activation and increased expression (Figure 3).^27^

#### 2.1.3 RET activation

Protein arginine *N*‐methyltransferase 2 (PRMT2) is an ERα co‐regulator, interacts with ERα66 and has the ability to inhibit breast cancer cell proliferation. Shen et al. found that after treating cells with tamoxifen, PRMT2 expression was reduced, ERα36 expression was increased, and tamoxifen resistance was mediated, while PRMT2 directly bound ERα36 and inhibited its activity, blocking PI3K/AKT and MAPK/ERK signalling pathway can reverse tamoxifen resistance.^28^ Shimoda et al^29^ found that aspartate‐β‐hydroxylase (ASPH) is related to the sensitivity of endocrine therapy. ASPH expression in tamoxifen‐resistant breast cancer cells was up‐regulated and MAPK and PI3K signalling pathways are involved in drug resistance regulation.

**FIGURE 4:**
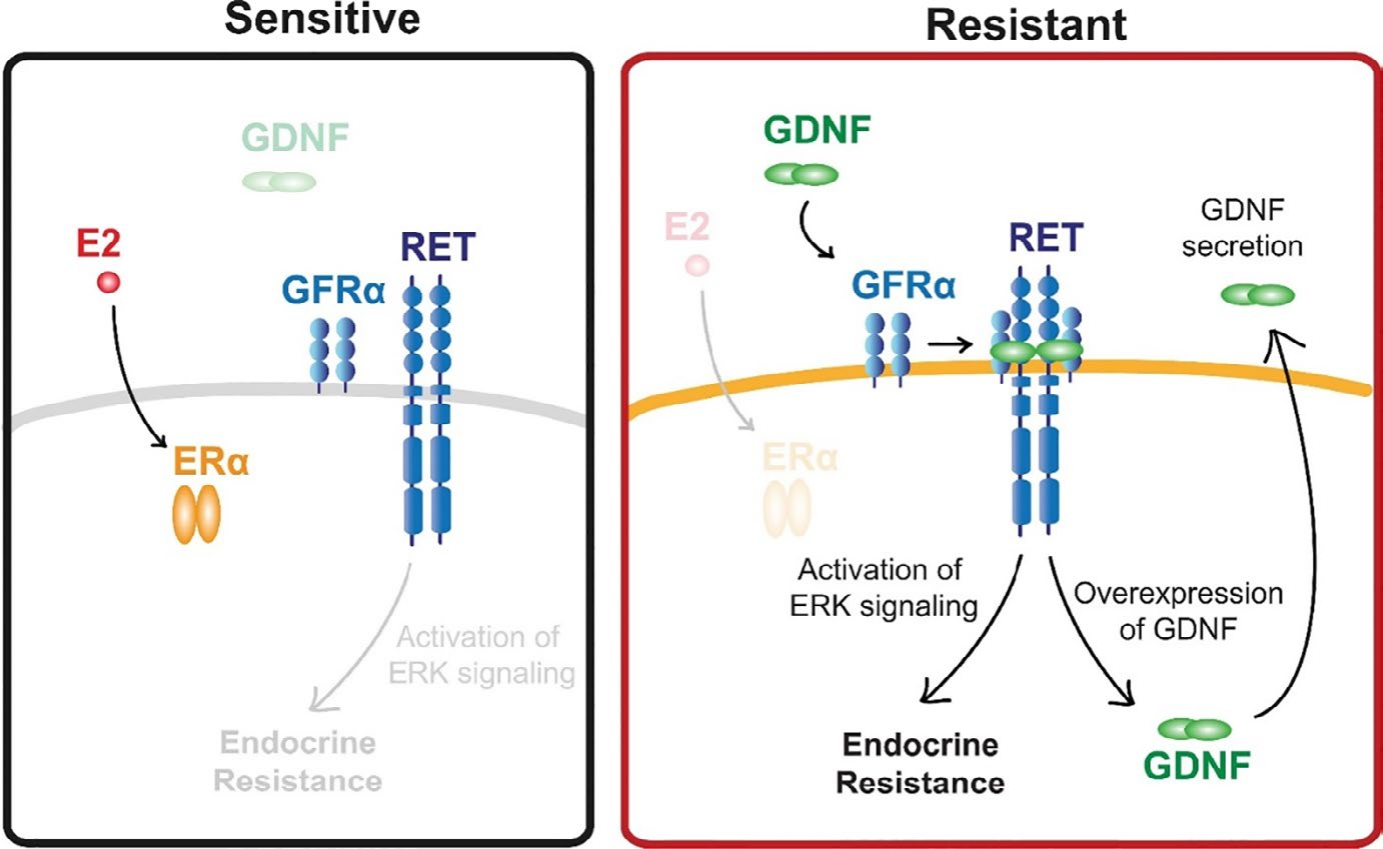
Schematic diagram of RET activation in endocrine sensitive and resistant tumours. Both endocrine sensitive and resistant breast cancer cells express all components of the RET signalling pathway, but endocrine sensitive breast cancer cells lack GDNF to initiate the resistance pathway. By contrast, endocrine resistant cells secret GNDF, which acts in an autocrine or paracrine fashion to promote endocrine resistance in nearby cells. Reprinted from [30]. Copyright © 2018, PLoS ONE

It has been well documented that ER‐positive breast cancers present the functional receptor tyrosine kinase RET signalling activity. Breast cancers that are sensitive to endocrine therapy often lack RET ligands. In support, the RET ligand GDNF has been shown to induce endocrine therapy resistance (Figure 4).^30^ Another study found that oestrogen directly activates C‐terminal Src kinase (CSK) expression in ER‐positive breast cancer, activates p21‐activated kinase2 (PAK2) and causes oestrogen‐independent growth. ER‐positive breast cancer with PAK2 overexpression is linked with resistance to endocrine therapy and poor prognosis. This study used PAK2 inhibitors and ER antagonists on drug‐resistant cells to synergistically inhibit breast cancer growth.^31^ Liang et al^32^ found that after 4‐OH‐tamoxifen treated MCF‐7 cells, melanoma cell adhesion molecule (MCAM) was resistant to tamoxifen‐resistant MCF‐7‐Tam‐R expression in cells is increased, and MCAM promotes tamoxifen resistance by activating the AKT signalling pathway, inducing epithelial–mesenchymal transition.

### 2.2 Genetic and epigenetic factors in endocrine therapy resistance

#### 2.2.1 Genetic factors

Somatic mutations in the ligand‐binding domain of oestrogen receptor 1 (ESR1) can cause resistance to endocrine therapy in breast cancer. E380Q, as a mutation of ESR1, is related to oestradiol (E2) hypersensitivity, the increased ability of DNA to bind to oestrogen response elements, and the activity of E2‐independent constitutive transactivation. Takeshita et al.^33^ tested ESR1 mutations (E380Q, Y537S, Y537N, Y537C and D538G) of tumour tissue and plasma cell‐free DNA (cfDNA) in 62 patients and found that 21% of patients with advanced breast cancer have ESR1 mutations. The incidence of mutations was 16% for E380Q, 41.6% for Y537S and 33.3% for D538G, Y537N and Y537C. Some samples detected double or triple mutations in ESR1. New anti‐estrogens (AE) targeting mutations have been found to have the ability to inhibit breast cancer cell proliferation and have effects on both wild‐type and Y537S and D538G mutants.^34^ Rinath et al. tested somatic ESR1 mutations in 76 metastatic ER^+^ breast cancer patients and found the frequency of these mutations was 12%. In preclinical models, these mutations were shown to bring about constitutive activity and relative resistance to endocrine therapy. They believe that these mutations are responsible for driving endocrine resistance in ER^+^ metastatic breast cancer.^35^ Keren et al. detected an ERα mutation (D538G substitution) in patients with metastatic breast cancer. This mutation leads to a conformational change in the ligand binding domain and further results in resistance to endocrine therapy.^36^

IGF1 is an important regulator of breast development and tumourigenesis. IGF1 and ERα interact in ESR1 mutant cells. Li et al. made ESR1 mutations (Y537S and D538G) in MCF‐7 and T47D cell lines through gene editing.^37^ The mutant cells showed IGF signal activation, and IGF1 was more sensitive to growth stimulation. The combined application of IGF1 receptor inhibitor (OSI‐906) and fulvestrant in mutant cells can synergistically inhibit tumour cell growth. Martin et al. first reported the existence of ESR1Y537C and ESR1Y537S mutations and found that cells have resistance to endocrine drugs after long‐term oestrogen deprivation.^38^ This naturally occurring ESR1 mutation will provide an important research model for the future basic studies of resistance mechanisms in breast cancer endocrine therapy.

Mutations in the CYP19A1 gene, which encodes a member of the cytochrome P450 enzyme superfamily, can result in increased or decreased aromatase activity. In a study by Magnani et al, they found that 21.5% of AI‐treated relapsed patients acquired CYP19A1 gene amplification. CYP19A1 amplification not only increased aromatase activity, but also led to oestrogen‐independent ERα binding to target genes, triggering CYP19A1^amp^ cells to show decreased sensitivity to AI treatment.^39^ They also found that some patients whose disease progressed after reversible AI treatment occasionally responded to irreversible AI.

Germline genetic variation can affect the risk and treatment outcome of breast cancer. Changes in SNPs (such as reduced ZNF423SNP and increased CTSOSNP) are associated with breast cancer risk. ER‐positive breast cancer CTSO affects the efficacy of tamoxifen by regulating the levels of ZNF423 and BRCA1. For patients with SNP (CTSO and ZNF423) associated with tamoxifen resistance, PARP inhibitors can reverse tamoxifen resistance.^40^

#### 2.2.2 Epigenetic factors

Non‐coding RNA (ncRNA) contains microRNA (miRNA) and long non‐coding RNA (lncRNA). miRNA regulates gene expression by inhibiting or degrading mRNA transcription through translation, and is involved in cell proliferation, differentiation, and apoptosis; lncRNA is involved in the intracellular protein backbone, chromatin circulation, and regulation of mRNA stability.^41^

It has been demonstrated that the expression of Mirna‐449a was significantly down‐regulated in tamoxifen‐resistant breast cancer cells and patients' peripheral blood. MiRNA‐449a regulates ADAM22 and further affects tamoxifen resistance through PPARG, LGI1, KRAS and LYN. Recovery of miRNA‐449a expression can reverse tamoxifen resistance.^42^ Another study reported that in endocrine‐sensitive breast cancer MCF‐7 cells, the expression of miRNA‐29a and miRNA‐29b‐1 was inhibited after tamoxifen was used, while miRNA was inhibited by tamoxifen in resistant cells LY2. Activated, tamoxifen can reduce the target of miRNA‐29 in LY2 cells, DIC‐ER1. Overexpression of miRNA‐29a and miRNA‐29b‐1 can reduce breast cancer cells' (MCF‐7, LCC9 and LY2) proliferation and inhibit LY2 cells' migration and tumour colony formation, but does not increase the sensitivity of LCC9 or LY2 cells to tamoxifen.^43^

Studies have found that HOTAIR in lncRNA affects the regulation of HOXD by affecting polycomb repressor complex 2 (PRC2) binding to homeobox D (HOXD) cluster DNA. PRC2 can promote the histone H3K27 trimethylation (H3K27me3), thereby inhibiting transcription, leading to blocked differentiation and increase the metastasis and invasion of breast cancer cells. HOTAIR overexpression is thought to be related to oestrogen response elements in its promoter, and E2 induces HOTAIR expression in breast cancer. HOTAIR is upregulated in tamoxifen‐resistant ER‐positive breast cancer and further causes tamoxifen resistance.^44^, ^45^ Wu et al.^46^ found that UCA1 in lncRNA enhanced breast cancer cells' resistance to tamoxifen by inhibiting the mTOR signalling pathway. UCA1 expression was significantly upregulated in tamoxifen‐resistant cells, and LCC2 and LCC9 cells transfected with UCA1 siRNA had a higher apoptotic rate after using tamoxifen. The study also found that UCA1 siRNA significantly reduced pAKT and p‐mTOR protein levels in LCC2 and LCC9 cells, and MCF‐7 cells over‐expressed UCA1 could reduce tamoxifen‐induced apoptosis. The protective effect of UCA1 on tumour cells was weakened after Rapamycin treatment.

**FIGURE 5:**
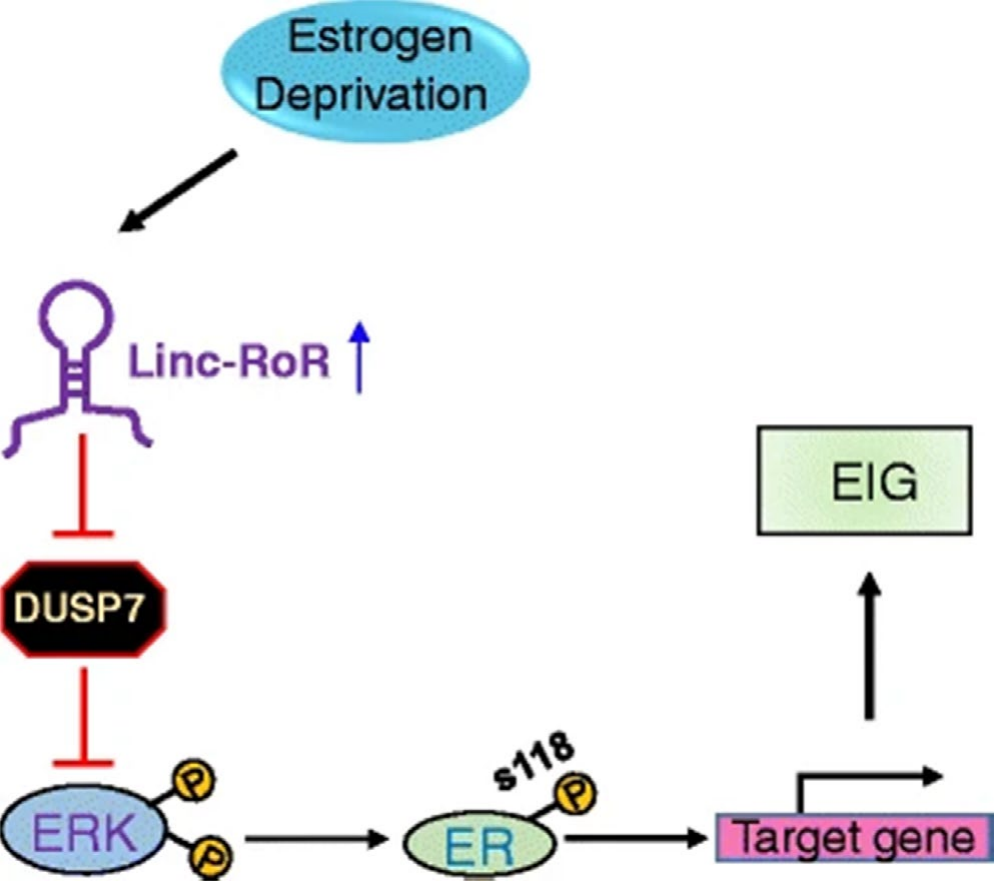
The working model of present study. Linc‐RoR promotes oestrogen‐independent growth (EIG) of ER^+^ breast cells through conferring the activation of MAPK/ERK pathway. See text for explanation. Reprinted from [447]. Copyright © 2017, Molecular Cancer volume

LincRNA‐regulator of reprogramming (Linc‐RoR), a regulator that promotes oestrogen‐independent breast cancer cell growth and tamoxifen resistance, plays an essential role in endocrine therapy resistance of breast cancer. Studies have found that Linc‐RoR expression is still up‐regulated after the cells were deprived of oestrogen, and knocking out Linc‐RoR can eliminate oestrogen‐independent growth of MCF‐7 cells and reverse the cell phenotype. At the same time, dual specificity phosphatase7 (DUSP7) is used as a negative regulator of ERK, which reduces the stability of DUSP7 through Linc‐RoR and activates the MAPK/ERK signalling pathway (Figure 5).^47^

### 2.3 Cancer stem cells and tumour microenvironment in endocrine therapy resistance

#### 2.3.1 Cancer stem cells

Cancer stem cells (CSCs), a rare tumour cell population, accumulate and likely contribute to their failure after anti‐oestrogen treatment. Several studies have reported enrichment of CSCs after endocrine therapies.^48^, ^49^ Endocrine therapies enhance CSCs' stem‐like feature and tumour‐initiating ability, which probably responsible for treatment resistance and subsequent disease progression.^50^, ^51^ Breast cancer stem cells have often been involved in endocrine resistance, although it is less clear whether they are enough to drive resistance. Simoes et al.^48^ reported that the activity and the frequency of breast cancer stem cell (BCSC) in ER^+^ patient samples and early and metastatic patient‐derived xenografts (PDXs) were increased after tamoxifen and fulvestrant endocrine therapy. Their study indicated that endocrine therapies do not target BCSCs, which may interpret how residual drug‐resistant cells cause the relapse of ER^+^ tumours after hormonal treatment. Tumours of patients with poorer outcomes after endocrine therapies revealed elevated ALDH1 expression and NOTCH4 activation. Moreover, endocrine therapy for ER^+^ breast cancer specifically increases the JAG1‐NOTCH4 signalling pathway, and its combination with the Notch pathway inhibitor can restrain BCSC enrichment leaded by endocrine therapies. Thus, JAG1 ligand signalling through the NOTCH4 receptor is a decisive factor for gaining endocrine resistance in ALDH‐positive cell populations. SRC3 can promote the stem‐like state of CSCs and induce EMT. It can also lead to worse outcomes and tamoxifen resistance of patients when overexpressed in breast cancer cells.^26^, ^52^ Other stem cell‐promoting factors such as SOX9 and FOXM1 are also involved in endocrine resistance.^53^, ^54^

#### 2.3.2 Tumour microenvironment

The tumour microenvironment (TME) refers to the surrounding microenvironment in which tumour cells exist, including surrounding blood vessels, fibroblasts, immune and inflammatory cells, signalling molecules and the extracellular matrix (ECM). A growing amount of evidence has suggested that several TME components such as cancer‐associated fibroblasts, ECM and immune and inflammatory cells play a pivotal role in endocrine resistance.

##### Hypoxia

Hypoxic microenvironments in solid tumours result from available oxygen being consumed by rapidly proliferating tumour cells, resulting in the oxygen levels around the tumour are significantly lower than in healthy tissues. Studies have also shown that hypoxia is related to endocrine resistance.^55^ Hypoxia‐inducible factor 1‐alpha (HIF‐1α) can induce cancer cell resistance to tamoxifen and fulvestrant treatment, and its over‐expression in ERα^+^ patients is associated with poor survival to endocrine therapy.^56^ Morotti et al. suggested that HIF‐1αmakes up for the deficiency of ERα expression for keeping up the expression of SNAT2 under hypoxia or endocrine treatments. In vivo, SNAT2 overexpression produces complete resistance to anti‐oestrogen therapy and induces tamoxifen resistance. Its expression is related to breast cancer patients' poor survival and resistance to endocrine therapy in ER‐receptor^+^ patients.^57^ There are also other studies demonstrating that hypoxia and HIF‐1α may play a significant role in endocrine treatment resistance.^55^, ^58^, ^59^

##### Cancer‐associated fibroblasts

Cancer‐associated fibroblast (CAF) is a cell type that, by initiating the extracellular matrix's remodelling or secreting cytokines, promotes tumourigenic features. CAFs provide pathways for aggressive cancers and promote invasion and metastasis by the biochemical alteration and cancer‐related pathways' regulation.^60^ CAFs are the largest stromal cell population in breast cancer and play a key role in breast cancer cells proliferation by producing cytokines and growth factors, remodelling of ECM and modulating immune cell function, further leading to endocrine resistance.^61^, ^62^ Brechbuhl et al. analysed the presence of CD146‐positive and CD146‐negative CAFs in ER^+^ breast cancer patients' tissues and found that CD146‐negative CAFs reduce ER expression and tumour cell sensitivity to oestrogen and tamoxifen sensitivity in ER breast cancer cells. They also demonstrated that CD146‐negative CAFs are associated with poor drug response to tamoxifen and worse outcome in patients.^63^

##### Extracellular matrix

The extracellular matrix (ECM) consists of extracellular macromolecules and minerals, and researchers have proved that it plays a crucial role in breast cancer progression and endocrine resistance.^64^ Jansen et al.^65^ examined 112 ER‐positive primary breast cancers with the advanced disease after treated with tamoxifen and clearly defined the type of treatment response (52 with objective response vs 60 with progressive disease). They identified 81 genes expressed differently between tamoxifen‐sensitive and drug‐resistant tumours. Forty‐four genes were extracted for predictive characteristics and verified on an independent group of 66 tumours. Among the identified genomes, a group of ECM genes, namely TIMP3, fibronectin 1 (FN1), LOX, collagen type 1 alpha 1 chain (COL1A1), SPARC and tenascin C (TNC), were found to be relevant to tamoxifen resistance. Pontiggia et al. showed that the factors produced by fibroblasts derived from M05 mouse breast tumours make tamoxifen resistant to other sensitive epithelial cells. On the other hand, fibronectin binds to β1 integrin and activates the MAPK/ERK1/2 and PI3K/AKT pathways, making epithelial cells refractory to tamoxifen. In conclusion, tumour stroma can lead to tamoxifen resistance. Combining endocrine therapy and treatment for the tumour microenvironment has the potential to be an alternative approach to conquer endocrine resistance in breast cancer.^66^

##### Extracellular vesicles

Extracellular vesicles (EVs), small membranous vesicles released by cells into the extracellular matrix, have been demonstrated to be potential modulators of tumour progression and drug resistance by translocating genetic material into recipient cells.^67^ Sansone et al.^68^ demonstrated that CAFs can package intact mitochondrial genome into EVs, which is released and absorbed by dormant CSCs, subsequently transcribing donor mtDNA, contributing to the restoration of oxidative phosphorylation (OXPHOS) and CSCs' self‐renovating and the endocrine therapy resistance. Therefore, we believe that EVs play a substantial role in endocrine therapy resistance.

##### The immune system

Inflammation coordinates the microenvironment surrounding the tumour and promotes proliferation, survival and migration.^69^ In recent studies, inflammatory cytokines have been correlated with endocrine resistance. Tumour‐associated macrophages (TAMs) are the main part of the tumour microenvironment in the majority of cancers. TAMs can strengthen breast cancer cell aggression by degrading ECM, promoting tumour angiogenesis and restraining cytotoxic T cells' anti‐tumour function, causing poor patient prognosis.^70^ Castellaro et al.^71^ found that animal xenograft tumours did not grow when MCF‐7 cells were injected in animal xenograft tumours after removal of oestradiol particles but grew when co‐injected with macrophages, suggesting that macrophages might promote breast cancer endocrine resistance.

Anti‐oestrogen treatment can induce transforming growth factor β (TGFβ) cytokines in breast cancer cells, resulting in the development of anti‐oestrogen resistance and immunosuppression.^72^ Stender et al. indicated that pro‐inflammatory cytokines such as IL‐1β and tumour necrosis factor α (TNFα) result in ERα‐dependent transcriptional activation by IKKβ‐dependent phosphorylation of ERα‐S305, contributing to endocrine resistance.^73^

The interplay between tumour cells and immune cells is increasingly pivotal in the development and progression of cancer. In a phase III trial, researchers examined the clinical relevance of tumour‐infiltrating lymphocytes (TILs) and breast cancer and found that TILs were lower in the ER‐positive/HER2‐negative subgroups compared with ER‐negative/HER2‐negative and HER2‐positive breast cancer subgroups (2.9% vs 10.6% and 11.1%).^74^

Rugo et al. analysed the programmed death ligand 1(PD‐L1)'s expression levels in ER^+^ tumours and only 20% of patients expressed PD‐L1, and single‐agent immune checkpoint inhibitors (ICIs) had limited efficacy in ER^+^, PD‐L1‐positive tumours.^75^ Another study investigated the relationship between ERα and PD‐L1 in breast cancer and identified ERα as a negative regulator of PD‐L1 gene transcription.^76^ Anurag et al. identified the upregulated genes in endocrine therapy resistant tumours via unbiased genome‐wide profiling analysis and showed that overexpression of the immune checkpoint components IDO1, LAG3 and PD1 was correlated with AI‐resistant proliferation in luminal B tumours. IDO1 was also related to poor survival prognosis in luminal B cases.^77^ Future studies are needed to elucidate the contribution of immune cells in endocrine resistance.

## 3 PRL IN ENDOCRINE THERAPY RESISTANCE

**FIGURE 6:**
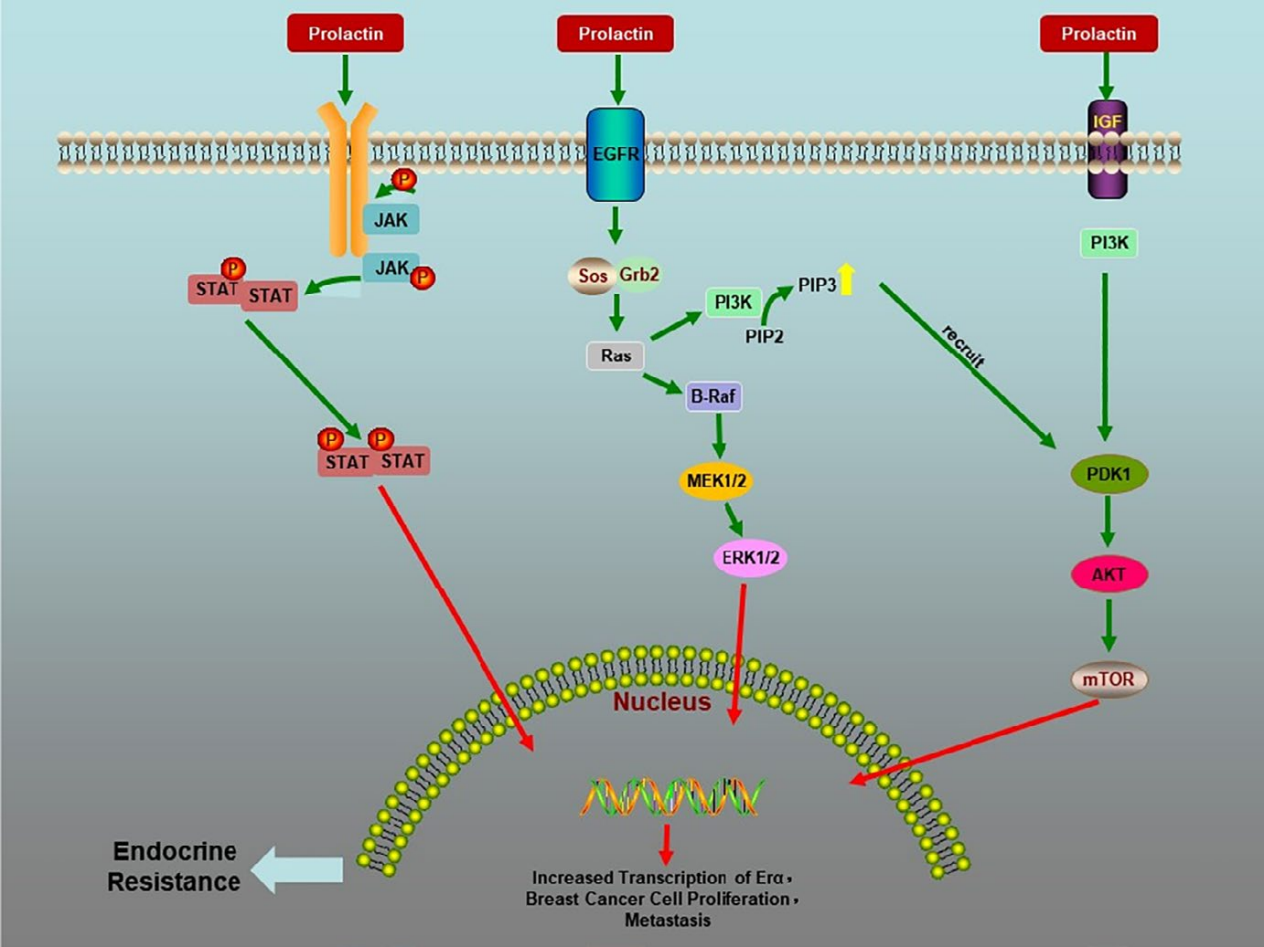
Prolactin in endocrine therapy resistance

Prolactin is a protein hormone secreted by adenohypophysis gland eosinophil. Its major function is to stimulate the growth of breast and induce and maintain milk lactation. Its secretion is modulated by hormones such as hypothalamic prolactin‐releasing inhibitory factor (PIF) and prolactin‐releasing factor (PRF), and can be self‐adjustment by short‐loop feedback. Recent studies focusing on prolactin's roles and mechanisms and its receptor in HR‐positive breast cancer were listed in Table 1. PRL participates in endocrine therapy resistance mainly by combining with PRLR and activating JAK2‐STAT5, ras‐raf‐ MEK‐ERK1 /2, Ras‐PI3K and other downstream signalling pathways. Figure 6 demonstrates the mechanism of prolactin in endocrine resistance.

**TABLE 1:** Recent studies focusing on the roles of prolactin and its receptor in HR‐positive breast cancer

Entry	Author	Year	Institution	Country	Journal	Key findings	PMID
1	Raghuveer Kavarthapu et al.	2016	Eunice Kennedy Shriver National Institute for Child Health and Human Development	USA	Oncotarget	1. EGF activation of EGFR through the intrinsic tyrosine kinase activity of the receptor and the activation of downstream signal transduction pathways (MAPK/ERK and PI3K/AKT), up‐ regulates the human prolactin receptor. Moreover, c‐SRC dependent EGF/EGFR induced events participate in this regulation 2. ERα and STAT5b in EGF/EGFR induced activation of PRLR gene transcription/expression in breast cancer cells via STAT5b interaction with ERα and complex formation with Sp1/C/EBPβ at the PRLR promoter in the absence of estrogen 3. The participation of the MAPK/ERK and PI3K/AKT pathways is required for phosphorylation of ERα and of c‐SRC/EGFRY845 in STAT5b phosphorylation for their recruitment to the PRLR promote	2.8E+07
2	Hamid H. Gar et al.	2016	Department of Pathology, University of Colorado School of Medicine	USA	Cancer Letters	1. AMPI‐109 treatment or knock down of PRL‐3 expres‐ sion were associated with deactivation of Src and ERK signaling and concomitant downregulation of RhoA and Rac1/2/3 GTPase protein levels. These cellular changes led to rearranged filamentous actin net‐ works necessary for cell migration and invasion 2. overexpression of PRL‐3 promoted TNBC cell invasion by upregulating matrix metalloproteinase 10, which resulted in increased TNBC cell ad‐ herence to, and degradation of, the major basement membrane component laminin 3. PRL‐3 engages the focal adhesion pathway in TNBC cells as a key mechanism for promoting TNBC cell migration and invasion	2.7E+07
3	Peter Oladimeji et al.	2016	The Department of Biological Sciences, University of Toledo	USA	Cancer Research	1. Estrogen activated PAK1 through both the ERa and GPER1 membrane receptors. Estrogen‐dependent activation of PAK1 required the phosphorylation of tyrosine residues 2. PKA RIIb subunit is a direct target of PAK1, the activated pTyr‐PAK1 complex reciprocally potentiated PKA activity. PKA phosphorylated Ser305‐ERa in response to estrogen, but pTyr‐PAK1 phosphorylated Ser305‐ERa in response to prolactin (PRL), implying that maximal ERa phosphorylation is achieved when cells are exposed to both PRL and estrogen 3. S305‐ERa activation led to enhanced phosphorylation of Ser118‐ERa and promoted cell proliferation and tumor growth 4. There is a critical interplay between PRL and estrogen via PAK1 and ligand‐independent activation of ERa through PRL/PAK1 may impart resistance to antiestrogen therapies	2.7E+07
4	Lynn N. Thomas et al.	2017	Departments of Biochemistry & Molecular Biology, Dalhousie University, Halifax, NS	Canada	Breast Cancer Res Treat	The CPD‐Arg‐NO pathway contributes to BCa progression in vitro and in vivo. PRL/androgen activation of the pathway support combined AR and PRLR blockade as an additional therapy for BCa	2.8E+07
5	Suzanne M. Schauwecke et al.	2017	Robert H. Lurie Comprehensive Cancer Center, Feinberg School of Medicine, Northwestern University	USA	The Journal of Biological Chemistry	1. Histone H1 prevents STAT5 binding at promoter DNA, and the PRL‐induced dissociation of H1 mediated by HMGN2 is necessary to allow full STAT5 recruitment and promote the biological effects of PRL signaling 2. The regulatory axis of HMGN2/H1 may serve as a target for future breast cancer therapeutics	2.8E+07
6	Katherine A. Leehy et al.	2018	University of Minnesota Masonic Cancer Center	USA	Journal of Steroid Biochemistry & Molecular Biology	1. PR and PRLR signaling cooperate in breast cancer 2. PR/PRLR pathways could offer alternative targets for breast cancer prevention	2.8E+07
7	Craig E. Barcus et al.	2016	Department of Comparative Biosciences, University of Wisconsin‐Madison, Madison	USA	Oncotarget	1. Both prolactin (PRL) and extracellular matrix (ECM) stiffness/density have been implicated in metastatic progression of Estrogen receptor a positive (ERa^+^) breast cancer 2. Culture of ERa^+^ breast cancer cells in dense/stiff 3D collagen‐I matrices shifts the repertoire of PRL signals, and increases crosstalk between PRL and estrogen to promote proliferation and invasion 3. Matrix stiffness shifts the balance of PRL signals from physiological (JAK2/STAT5) to pathological (FAK/SFK/ERK1/2) by increasing PRL signals through focal adhesions 4. PRL signaling to FAK and SFKs may be useful targets in clinical aggressive ERa^+^ breast carcinomas	2.7E+07
8	Alan Hammer and Maria Diakonova	2016	Department of Biological Sciences, University of Toledo	USA	BMC Cell Biology	Tyrosyl phosphorylation of PAK1 by PRL increases breast cancer cell metastasis in vivo	2.8E+07
9	Tyler M MacDonald et al.	2019	Departments of Biochemistry & Molecular Biology, Dalhousie University, Halifax	Canada	Am J Cancer Res	1. EDD levels increase with BCa progression in vivo 2. PRL‐inducible EDD in BCa cells promotes TORC1 signaling, anti‐apoptotic protein expression, and drug resistance in vitro	3.1E+07
10	Karolina Jablonska et al.	2016	Department of Histology and Embryology, Wroclaw Medical University, Wroclaw	Poland	Am J Cancer Res	1. Prolactin‐Induced Peptide (PIP) as a single gene differentially expressed in BC therapy responder or non‐responder patients (*p* < 0.05) 2. The expression of PIP mRNA and protein was higher in ER^+^ and PR + BC than in TNBC cases 3. Microarray analysis characterized PIP gene as a candidate for BC standard chemotherapy response marker. PIP as a factor differentiating patients responding to cyclophosphamide and doxorubicin chemotherapy	2.7E+07

### 3.1 JAK/STAT pathway

The physiological function of PRL is mainly mediated by PRLR‐JAK/STAT5 signalling pathway. PRL can bind to target cells' specific transmembrane receptors and activate the receptor‐related Janus tyrosine kinase (Jak)2. The activation of JAK can catalyse phosphorylation of tyrosine residues on the receptor. JAK kinase can recruit and phosphorylate the STAT protein on the receptor. The activated STAT protein enters the nucleus as a dimer to combine with the target gene, regulates gene transcription and increases ER transcription. PRL enhances oestrogen sensitivity, promotes tumour differentiation and reduces therapeutic sensitivity through this mechanism.

The conjugation of prolactin to the prolactin receptor (PRLR) activates STAT5a in the breast to facilitate the gene transcription involved in lobule development. A previous study showed that STAT5a expressed in majority of normal breast epithelial cells, but not generally in carcinoma in situ or invasive carcinoma, except in those secretory carcinomas that maintain STAT5a. Previous immunohistochemical studies have shown that PRLR is only expressed occasionally on the luminal surface of normal breast epithelial cells that express the STAT5a protein and in cells that express STAT5a protein and in cells that exhibit secretory changes in STAT5a expression, whether benign or malignant.^78^, ^79^ STAT5a and PRLR exhibit reciprocal expression patterns between normal and abnormal breast tissue. In DCIS and invasive carcinoma, reverse expression occurs in normal cells, causing an increase in PRLR and a decrease in STAT5a, which might indicate a disturbance in the signalling pathway that may be associated with carcinogenesis.^78^, ^79^

### 3.2 MicroRNA‐339‐5p‐dependent pathway

Studies have found that micro RNA339‐5p (miR‐339‐5p) target the gene B‐cell lymphoma 6 (BCL6) and the expression of BCL6 protein was related to the breast cancer progression.^80^ Hong Yan et al. revealed that PRL could inhibit BCL6 expression in human breast cancer cells and affect breast cancer development via the miR‐339‐5p‐dependent pathway.^80^ The study showed that PRL inhibited the expression of BCL6 and upregulated the expression of miR‐339‐5p in breast cancer cells. PRL‐induced suppression of BCL6 expression was dramatically reversed by selective downregulation of miR‐339‐5p expression. Exogenous PRL stimulation remarkably reduced the proliferation, colony formation, metastasis, and invasion of breast cancer cells, while miR‐339‐5p expression's suppression reversed these processes in vitro.^80^

### 3.3 Prolactin receptor signalling pathway

Calcium‐modulating cyclophilin ligand (CAML) is a signalling protein recognized by the TNF receptor TACI (transmembrane activator and CAML‐interactor). CAML is also involved in signalling at other receptors, such as the EGFR^81^and mucin 1 (MUC1).^82^ Ji‐Hong Lim et al. found high expression of CAML in breast cancer cells and tissues.^83^ Moreover, CAML was found to bind to PRLR, required for PRLR function, such as activation of Stat5 and Mek1/2, internalizing PRL with cyclophilin B, recycling of PRLR, and increasing Ca(2+) mobilization. CAML also interacts with PRLR and is indispensable for the PRL‐induced activation of PRLR signalling. In addition, CAML is essential in the growth of PRL/PRLR‐dependent breast cancer cells.^83^ These findings indicate that CAML is thought to be a promising target for controlling breast cancer progression.

### 3.4 Ras‐Raf and Ras‐PI3K signalling pathway

Prolactin can cooperate with IGF‐I and EGF family ligands to combine with receptor tyrosine kinase (RTK) on the surface of cell membrane in the absence of oestrogen, after which RTK activates two critical signalling molecules through its tyrosine kinase activity: Small G protein Ras and kinase PI3K. Next, downstream key molecule mammalian target of rapamycin (mTOR) are jointly activated by Ras and PI3K. The activated mTOR protein promotes substrate phosphorylation and gene transcription, activates the non‐ligand‐dependent transcription activation function 1 (AF‐1), induces phosphorylation of ERα and promotes proliferation and invasion of breast cancer cells, causing endocrine therapy resistance. Ras‐Raf‐ERK1/2 pathway can also directly induce transcription.

#### 3.4.1 Ras‐Raf signalling pathway

Prolactin can initiate the phosphorylation of HER2. The study of Knuefermann et al. proved that the PI3K/AKT signalling pathway activated by HER2 plays an important role in the multidrug resistance (MDR) of breast cancer cells.^84^ Meanwhile, PRL activates RAF by activating Ras protein. When Raf is activated, its C‐terminal catalytic region can combine with MEK and phosphorylate the 2 Ser kinases in the eighth subregion of its catalytic domain, activating MEK. The activated MEK directly connects with ERKs through its N‐terminal region, activating ERK. The ERK includes ERK1 and ERK2. The ERK1/2 activated by phosphorylation enters the cell nucleus through the cytoplasm and participates in cell proliferation and differentiation, cell carcinogenesis and other biological reactions.

#### 3.4.2 Ras‐PI3K signalling pathway

**FIGURE 7:**
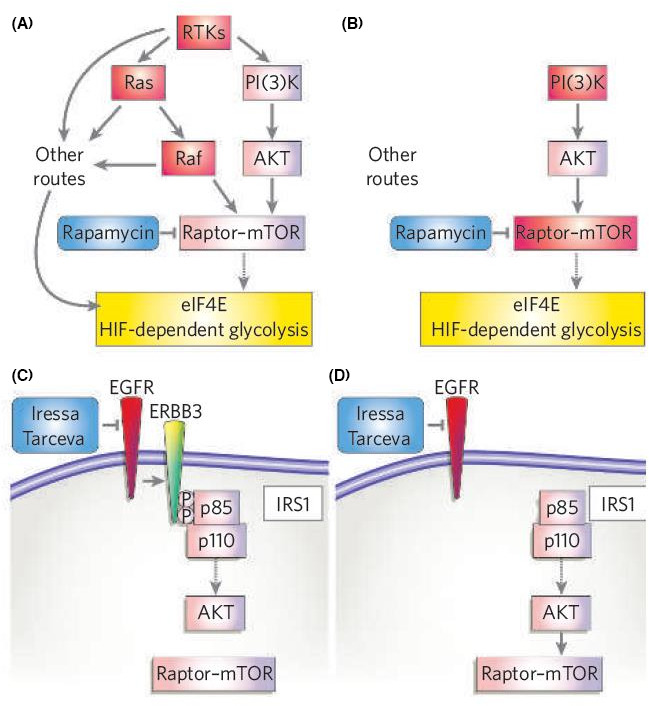
Pathway circuitry dictates therapeutic response. (A) For tumours with defined genetic lesions, the ability to overcome a given targeted therapeutic lies in whether or not they need to acquire a secondary genetic mutation to overcome the effect of the drug on critical downstream biochemical effectors that are required for continued tumour cell growth, or whether they can simply upregulate existing alternative routes that lead to effectors already expressed in those cells. So, the drug places selection pressure to ramp up existing bypass routes. If there are no such routes to the critical downstream effectors, a specific mutation to upregulate those alternative routes or bypass the drug are required. In this example, a critical target for tumour cell growth and survival is the activation of eIF4E and HIF. Tumours with initiating mutations in RTKs, Ras or Raf have multiple routes to signal to eIF4E and HIF, so blocking mTOR with rapamycin does not inhibit these tumours. (B) In contrast, tumours with initiating lesions in PI(3)K or more direct regulators of mTOR (such as LKB1 and TSC) do not have alternative routes to activate eIF4E and HIF. Hence these tumours show greater response to rapamycin. (C) Similarly, the expression and use of specific adaptor proteins that enhance certain arms of pathway signalling will dictate the therapeutic response. In the example shown, human lung tumours expressing epidermal‐growth‐factor receptor (EGFR) are targeted with anti‐EGFR drugs such as Iressa or Tarceva. In tumours expressing the ERBB3 heterodimerization partner, EGFR efficiently enhances PI(3)K activation through a number of PI(3)K‐binding sites in ERBB3. (D) In tumours that lack ERBB3, PI(3)K is still activated by a number of other routes, including adaptors such as insulin receptor substrate 1 (IRS1). Reprinted from [86]. Copyright© 2006, Nature

As early as in 1998, Yamauchi et al. found in the cells transfected by PRLR that PRL phosphorylates insulin receptor substrate (IRS).^85^ This is relevant to the subunit regulation of PI3K kinase, which further activates protein kinases B (PKB or AKT) as a second messenger by generating phosphatidylinositol 3‐phosphate (PI3P). PKB or AKT is an essential downstream molecule of PI3K, plays an essential role in regulating cell growth, proliferation, survival and glycose metabolism of cells and PI3K/AKT pathway can activate the downstream target protein mTOR (Figure 7).^86^ mTOR protein is an important regulator of cell growth and proliferation. The activated mTOR protein promotes the phosphorylation of key regulators in protein translation, leading to the increase of ribosomal protein synthesis. The enhancement of PI3K/AKT signalling pathway is one of the causes of hormonal resistance in breast cancer. Tokunaga et al.^87^ proved that activation of Akt is essential to induce endocrine therapy resistance in breast cancer.

#### 3.4.3 Ras‐Raf and Ras‐PI3K signalling pathways interact

PI3K/AKT pathway is required in activating MAPK/ERK pathway by PRL.^88^ PI3K can directly phosphorylate P‐Rex1 enzyme, activate ras‐related c3 botulinum toxin substrate 1 (Rac1) and form Rac‐GTP, further activate p21‐activated kinase (PAK), which can directly activate Raf/MEK/ERK.^89^ Therefore, PI3K can regulate MEK/ERK bypassing AKT. Except for playing a role via Raf/MEK/ERK pathway, PAK1 can also phosphorylate ER and promote its transcription, thus up‐regulating the expression of cyclin D1 and promoting hormone‐dependent breast cancer cell growth. It may also promote angiogenesis by upregulating VEGF expression, which is conducive to tumour metastasis.^90^ In the study of breast cancer cells without metastatic potential, the overexpression of continuously activated PAK1 promotes cell migration and proliferation. PRL antagonizes tamoxifen by inducing PAK1 to cooperate with the overexpression of ER.^91^

#### 3.4.4 FAK and Scr kinase families

**FIGURE 8:**
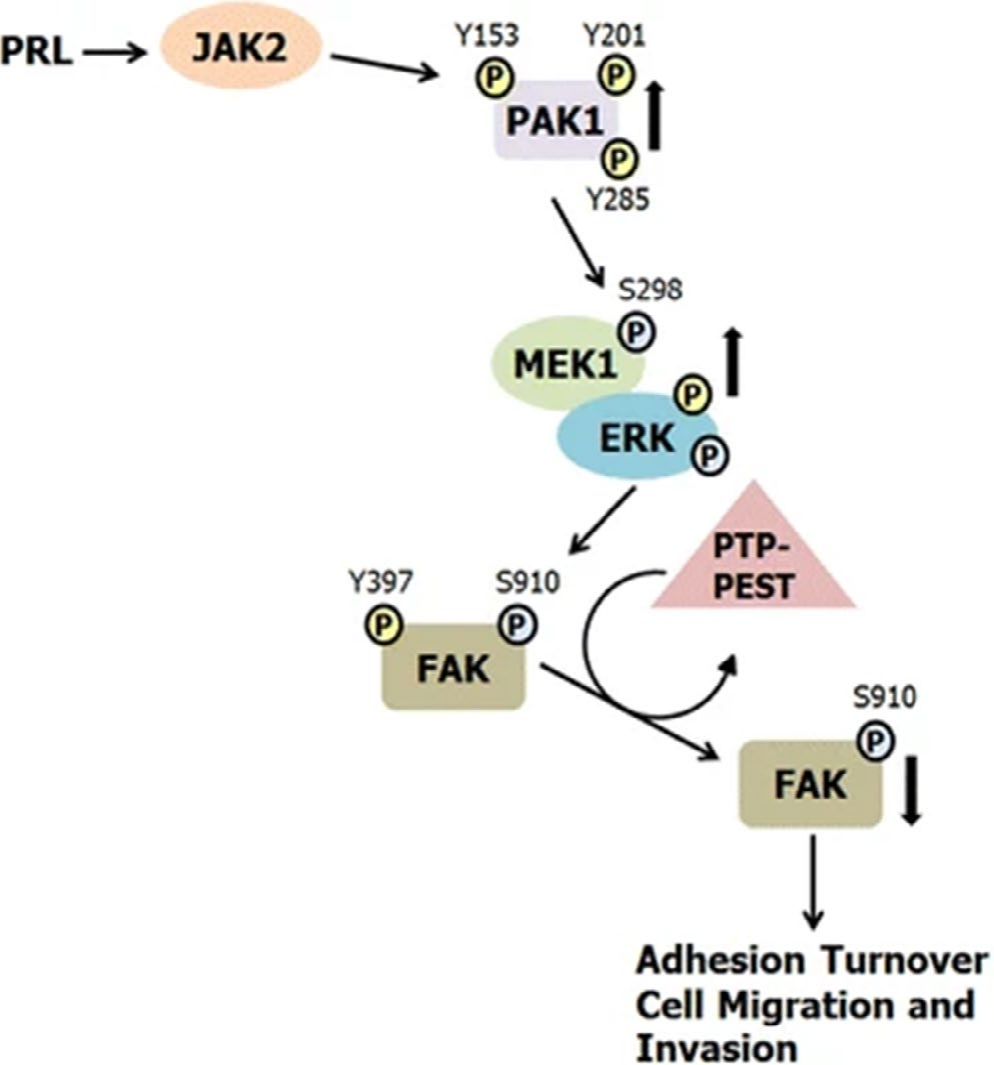
Proposed mechanism for the role of PRL‐activated PAK1 in breast cancer cell migration. PRL binding to the PRLR results in activation of the non‐receptor tyrosine kinase JAK2. JAK2 tyrosyl phosphorylates PAK1 on Y153, 201, and 285, enhancing PAK1 kinase and scaffolding activities. PRL treatment also leads to FAK auto‐phosphorylation at Y397. Activated PAK1 phosphorylates MEK1 at S298, resulting in increased MEK1/ERK binding and enhanced ERK activity. Active ERK phosphorylates FAK at S910, leading to dephosphorylation of FAK at Y397 by the tyrosine phosphatase PTP‐PEST as shown by Zheng et al. (2009). FAK dephosphorylation decreases FAK kinase activity and promotes adhesion turnover and breast cancer cell migration. Reprinted from [92]. Copyright © 2016, BMC Cell Biology

The development of breast cancer is largely dependent on cell movement and the changeability of cytoskeletal actin. The studies in recent years showed that actin needs to combine with cell membrane. Next, focal adhesion kinase (FAK) is required to mediate the strong adhesion of the extracellular matrix (Figure 8). These processes are regulated by c‐Src.^92^ The effect of PRL stimulates the expression and phosphorylation of c‐Src and FAK in breast cancer cells and enhances the movement of breast cancer cells. This finding expanded the understanding of the effect of PRL on breast cancer cells and suggested a new pathway in which it is relevant to breast cancer progression.

## 4 ADVANCES IN ENDOCRINE THERAPY RESISTANCE

With the proposal of more and more endocrine therapy resistance mechanisms, more drugs can be used for the treatment of endocrine resistance. For instance, the increased activity of AKT/mTOR pathways is the main mechanism of the resistance to letrozole and flurvesant; and the mTOR inhibitor everolimus can block PI3K/AKT/mTOR pathway. Preclinical studies showed that the median overall survival (OS) in tamoxifen + everolimus and exemestane + everolimus groups is longer than that in the control group and that the combination of mTOR inhibitor and endocrine therapy can be used as a therapy for patients with aromatase inhibitor resistance.^93^

Cyclin‐dependent kinase (CKD) is a new target for treating breast cancer. CDK4 /6 inhibitor arrests cell cycle. The FDA has approved Palbociclib and ribociclib as first‐line therapy for advanced hormone receptor‐positive breast cancer. TGF‐β type 2 receptor impairs the oestrogen response and gives tamoxifen resistance.^94^ The dual inhibitors for TGF‐β and MAPK are being studied. Such inhibitors may be effective in patients with endocrine therapy resistance.^95^ Palbociclib combined with fulvestrant is an effective therapy for endocrine resistance in advanced premenopausal breast cancer (HR^+^/HER2^−^).^96^, ^97^ The US Food and Drug Administration has approved fulvestrant for treating metastatic breast cancer in hormone receptor‐positive postmenopausal women with disease progression after anti‐oestrogen therapy.^98^ The BELLA‐2 study showed that postmenopausal women who developed disease progression during or after treatment with an aromatase inhibitor for HR‐positive/HER2‐negative advanced breast cancer had a significant improvement in PFS in patients after buparlisib plus fulvestrant.^99^ Similarly, the phase III BELLA‐3 study showed that buparlisib plus fulvestrant improved PFS in HR‐positive, HER2‐negative, locally advanced or metastatic breast cancer patients who progressed after being treated with everolimus plus exemestane.^100^ HDAC inhibitors are also potential drugs to reverse endocrine resistance in HR‐positive breast cancer. Entinostat is an oral selective HDAC inhibitor that restores patients' sensitivity to AIs by upregulating ER receptor and aromatase enzyme levels.^101^ The phase II ENCORE 301 study has shown that entinostat plus exemestane improved PFS and OS vs exemestane monotherapy in postmenopausal patients with HR‐positive advanced breast cancer and prior non‐steroidal AI therapy progression.^102^

PRL can induces PAK1. As a common PRL and oestradiol (E2) pathway, PAK1 cooperates with ER to stimulate tumour cell metastasis, inducing tamoxifen resistance. Argentine Molecular Oncology Laboratory has developed specific Rac1 inhibitor 1A‐116, which inhibits the activity of PAK1 and restores the drug sensitivity of tamoxifen‐resistant cells.^103^ Studies showed that the reduction of EGR1 protein level can cooperate with anti‐oestrogen to inhibit the proliferation of breast cancer cells; and EGR1 is expected to be an important target to prevent or reverse endocrine resistance.^104^

The PRL secreted by breast cells cannot be controlled by the dopamine agonist acting on the pituitary gland level. It is necessary to develop a particular type of drug to block PRLR to reduce endogenous PRL. Howell et al.^105^ discovered that PRLR antagonist Δ1–9‐G129R‐hPrl hinders the activation of PRLR, which promotes the apoptosis of ER^+^ and ER^−^ breast cancer cells, increases the cytotoxic effect of doxorubicin and paclitaxel in vitro. They also proposed that the reasonable combination of cytotoxic drugs and Δ1–9‐G129R‐hPrl can improve the therapeutic effect. American Huntsman Institute found, in the treatment of 73 patients with metastatic breast cancer (MBC) or metastatic castrate‐resistant prostate cancer (mCRPC) with LFA102 generated with PRLR inhibitor and monoclonal antibody, that LFA102 showed no antitumour activity as a monotherapy.^106^

Studies showed that the new PRLR antibody drug conjugates (ADCs) rapidly release the cytotoxic drugs carried by ADCs into the intracellular lysosomes; and the active drugs released can also infiltrate from target cells into adjacent antigen‐negative cells, which may cause the so‐called bystander killing. The results showed that ADCs have antitumour activity in vivo and in vitro.^107^ Some recently completed and ongoing clinical trials on breast cancer treatment targeting prolactin and its receptors were listed in Table 2.

**TABLE 2:** Some recently completed and ongoing clinical trials on breast cancer treatment targeting prolactin and its receptors

Entry	Drug	NCT Number	Trial title	Status	Conditions	Interventions	Study type	Study phase	Study design	Enrollment	Age	Sex	Sponsor/collaborators
1	LFA102	NCT01338831	Phase I study of LFA102 in patients with prolactin receptor‐positive castration‐resistant prostate cancer or prolactin receptor‐positive metastatic breast cancer	Completed	Castration‐resistant prostate cancer Metastatic breast cancer Uterine leiomyoma	Drug: LFA102	Interventional	Phase 1	Allocation: non‐randomized Intervention model: single group assignment Masking: none (open label) Primary purpose: treatment	73	18 years and older (adult, older adult)	All	Novartis Pharmaceuticals Novartis
2	cabergoline	NCT01730729	Cabergoline in metastatic breast cancer	Completed	Recurrent breast cancer Stage IV breast cancer	Drug: cabergoline	Interventional	Early phase 1	Intervention model: single group assignment Masking: none (open label) Primary purpose: treatment	20	18 years and older (adult, older adult)	Female	Northwestern University Lynn Sage Foundation
3	–	NCT00842465	Prolactin Receptor and Breast Diseases	Completed	Benign breast disease Breast cancer	Biological: blood collection for hormonal status analysis Procedure: breast Biopsy or surgery Genetic: blood collection Other: ultrasonography (pelvis and breast), bone mineral density	Observational	–	Time perspective: cross‐ sectional	735	10 years and older (child, adult, older adult)	Female	Assistance Publique ‐ Hôpitaux de Paris Institut Pasteur
4	Nimesulide Simvastatin Placebo	NCT01500577	A prevention trial in subjects at high risk for breast cancer	Completed	Breast cancer	Drug: nimesulide Drug: simvastatin Other: placebo	Interventional	Phase 2	Allocation: randomized Intervention model: parallel assignment Masking: double (participant, investigator) Primary purpose: prevention	150	18 years to 65 years (adult, older adult)	Female	European Institute of Oncology
5	LFA102	NCT01610050	A phase I study of LFA102 in Japanese patients	Completed	Castration‐resistant prostate cancer, advanced breast cancer	• Drug: LFA102	Interventional	Phase 1	Allocation: non‐ randomized Intervention model: single group assignment Masking: none (open label) Primary purpose: treatment	14	18 years and older (adult, older adult)	All	Novartis Pharmaceuticals Novartis
6	–	NCT00516698	Changes in breast density and blood hormone levels in postmenopausal women receiving anastrozole or exemestane for breast cancer	Completed	Breast cancer	Genetic: polymorphism analysis Other: high performance liquid chromatography Other: laboratory biomarker analysis Procedure: radiomammography	Observational	–	Observational model: case‐only Time perspective: prospective	140	18 Years and older (Adult, Older Adult)	Female	Alliance for clinical trials in oncology National Cancer Institute (NCI)
7	‐	NCT00973557	The effect of monoclonal vascular endothelial growth factor (VEGF) antibody (bevacizumab) on pituitary function	Completed	Colorectal cancer Lung cancer Breast cancer Glioblastoma	–	Observational	–	Observational model: cohort Time perspective: cross‐ sectional	6	18 years and older (adult, older adult)	All	Cedars‐Sinai Medical Center
8	–	NCT00860886	Premenopausal hormone concentrations in a population of women at very low risk of breast cancer	Completed	Menopause Normal physiology	–	Observational	–	Observational model: case‐control Time perspective: cross‐ sectional	433	25 years to 44 years (adult)	Female	National Cancer Institute (NCI) National Institutes of Health Clinical Center (CC)
9	Drug: GDC‐9545 Drug: Palbociclib Drug: LHRH agonist	NCT03332797	A study of GDC‐9545 alone or in combination with palbociclib and/or luteinizing hormone‐ releasing hormone (LHRH) agonist in locally advanced or metastatic estrogen receptor‐positive breast cancer	Recruiting	• Breast cancer	Drug: GDC‐9545 Drug: palbociclib Drug: LHRH agonist	Interventional	Phase 1	Allocation: non‐ randomized Intervention model: sequential assignment Masking: none (open label) Primary purpose: treatment	220	18 years and older (adult, older adult)	Female	Genentech, Inc.
10	Exemestane	NCT00073073	Exemestane and Celecoxib in Postmenopausal Women at High Risk for Breast Cancer	Completed	• Breast Neoplasms	Drug: exemestane Dietary supplement: Calcium carbonate Dietary supplement: vitamin D	Interventional	Phase 2	Intervention model: single group assignment Masking: none (open label) Primary purpose: prevention	46	Child, adult, older adult	Female	Georgetown University National Cancer Institute (NCI)
11	Drug: tamoxifen Drug: tamoxifen and GnRH analogue Drug: exemestane and GnRH analogue	NCT01638247	Tamoxifen +/− GnRH analogue vs aromatase inhibitor + GnRH analogue in male breast cancer patients	Completed	Male breast cancer	Drug: tamoxifen Drug: tamoxifen and GnRH analogue Drug: exemestane and GnRH analogue	Interventional	Phase 3	Allocation: randomized Intervention model: parallel assignment Masking: none (open label) Primary purpose: treatment	56	18 years to 85 years (adult, older adult)	Male	German Breast Group Pfizer
12	Drug: intravenous morphine sulphate Drug: intravenous tramadol HCL Drug: intravenous ketorolac tromethamine	NCT02449954	Effect of morphine, tramadol, and ketorolac on postoperative stress and immune responses	Unknown status	Breast cancer	Drug: intravenous morphine sulphate Drug: intravenous tramadol HCL Drug: intravenous ketorolac tromethamine	Interventional	Phase 2 Phase 3	Allocation: randomized Intervention model: crossover assignment Masking: double (investigator, outcomes assessor) Primary purpose: diagnostic	60	20 years to 60 years (Adult)	Female	Assiut University
13	Drug: dexmedetomidine injection [Precedex] Drug: bupivacaine	NCT03063073	Efficacy and safety of dexmedetomidine added to modified pectoral's block	Unknown status	Breast cancer female	Drug: dexmedetomidine injection [Precedex] Drug: bupivacaine	Interventional	Phase 3	Allocation: randomized Intervention model: parallel assignment Masking: triple (participant, care provider, outcomes assessor) Primary purpose: prevention	60	18 years to 60 years (Adult)	Female	South Egypt Cancer Institute
14		NCT02197000	A nutritional intervention to decrease breast density among female BRCA (BReast CAncer susceptibility gene) carriers	Unknown status	BRCA1 gene mutation BRCA2 gene mutation	• Dietary supplement: DIM‐ Avail 100 mg	Interventional	–	Intervention model: single group assignment Masking: none (open label) Primary purpose: prevention	36	18 years to 70 years (adult, older adult)	Female	Rabin medical center
15	ABBV‐176	NCT03145909	A study evaluating the safety, pharmacokinetics and anti‐tumor activity of ABBV‐176 in subjects with advanced solid tumors likely to express prolactin receptor (PRLR)	Terminated	Advanced solid tumors cancer	Drug: ABBV‐176	Interventional	Phase 1	Allocation: non‐ randomized Intervention model: parallel assignment Masking: none (open label) Primary purpose: treatment	19	18 years and older (adult, older adult)	All	AbbVie
16	–	NCT00006368	Yttrium Y 90 SMT 487 in treating patients with refractory or recurrent cancer	Completed	Brain and central nervous system Tumors Breast cancer Gastrointestinal carcinoid tumor Islet cell tumor Lung cancer Lymphoma Melanoma (Skin) Neoplastic Syndrome	Radiation: yttrium Y 90‐edotreotide	Interventional	Phase 1	Primary purpose: treatment	60	18 years and older (Adult, Older Adult)	All	Novartis Pharmaceuticals Novartis
17	Telapristone acetate	NCT02314156	Transdermal or Oral Telapristone Acetate in Treating Patients Undergoing Mastectomy	Active, not recruiting	BRCA1 mutation carrier BRCA2 mutation carrier Ductal breast carcinoma in situ Lobular breast carcinoma in situ Stage 0 breast cancer Stage IA breast cancer Stage IB breast cancer Stage IIA breast cancer Stage IIB breast cancer	Drug: telapristone acetate Other: placebo Other: laboratory biomarker analysis Other: questionnaire administration	Interventional	Phase 2	Allocation: randomized Intervention model: parallel assignment Masking: single (participant) Primary purpose: prevention	67	18 years and older (adult, older adult)	Female	Northwestern University National Cancer Institute (NCI)

## 5 CONCLUSION

In conclusion, PRL is closely correlated to the tumourigenesis, development and endocrine therapy resistance of breast cancer. Therefore, inhibiting PRL can enhance the sensitivity of breast cancer cells to drugs. However, there are no clinical data on whether the reduction of PRL level affects the patients with endocrine resistance in breast cancer. Currently, PRL targeted therapies are still at the experimental stage and face many challenges before real clinical application. Therefore, keep exploring the role of PRL and its targeted therapies in the mechanism of endocrine therapy resistance in breast cancer plays a significant role in its becoming a method to overcome endocrine therapy resistance clinically. It is likely to become a very promising new therapeutic target for endocrine therapy for breast cancer.

## CONFLICT OF INTEREST

The authors declare that they have no competing interests.

## AUTHOR CONTRIBUTIONS

**Yuan Li:** Writing‐original draft (equal). **Xiangyi Kong:** Writing‐review & editing (equal). **Lixue Xuan:** Supervision (equal). **Zhongzhao Wang:** Supervision (equal). **Yen‐Hua Huang:** Supervision (equal).

## Data Availability

Data sharing not applicable to this article as no datasets were generated or analysed during the current study.

## References

[1] Leon‐Ferre RA , Polley MY , Liu H , et al. Impact of histopathology, tumor‐infiltrating lymphocytes, and adjuvant chemotherapy on prognosis of triple‐negative breast cancer. Breast Cancer Res Treat. 2018;167(1):89‐99. 28913760 10.1007/s10549-017-4499-7 PMC5790598

[2] McGuire WL , Osborne CK , Clark GM , Knight WA . Steroid hormone receptors and carcinoma of the breast. Am J Physiol Endocrinol Metab. 1982;243(2):E99‐E102. 10.1152/ajpendo.1982.243.2.E99 7114210

[3] Clevenger CV , Chang WP , Ngo W , Pasha TL , Montone KT , Tomaszewski JE . Expression of prolactin and prolactin receptor in human breast carcinoma. Evidence for an autocrine/paracrine loop. Am J Pathol. 1995;146(3):695‐705. 7534043 PMC1869171

[4] Reynolds C , Montone KT , Powell CM , Tomaszewski JE , Clevenger CV . Expression of prolactin and its receptor in human breast carcinoma. Endocrinology. 1997;138(12):5555‐5560. 9389544 10.1210/endo.138.12.5605

[5] Mujagic Z , Mujagic H , Prnjavorac B . Circulating levels of prolactin in breast cancer patients. Med Arh. 2005;59(1):33‐35. 15822682

[6] Swaminathan G , Varghese B , Fuchs SY . Regulation of prolactin receptor levels and activity in breast cancer. J Mammary Gland Biol Neoplasia. 2008;13(1):81‐91. 18204982 10.1007/s10911-008-9068-6 PMC2276629

[7] He W . Expression Profiles and Regulatory Networks of Prolactin RECEPTOR‐related Micrornas in Breast Cancer. Department of Genetics, Nanjing Medical University; 2013.

[8] Tworoger SS , Eliassen AH , Zhang X , et al. A 20‐year prospective study of plasma prolactin as a risk marker of breast cancer development. Cancer Res. 2013;73(15):4810‐4819. 23783576 10.1158/0008-5472.CAN-13-0665 PMC3738582

[9] Tikk K , Sookthai D , Johnson T , et al. Circulating prolactin and breast cancer risk among pre‐ and postmenopausal women in the EPIC cohort. Ann Oncol. 2014;25(7):1422‐1428. 24718887 10.1093/annonc/mdu150

[10] Abech DD , Moratelli HB , Leite SC , Oliveira MC . Effects of estrogen replacement therapy on pituitary size, prolactin and thyroid‐stimulating hormone concentrations in menopausal women. Gynecol Endocrinol. 2005;21(4):223‐226. 16316844 10.1080/09513590500279717

[11] Foth D , Romer T . Prolactin serum levels in postmenopausal women receiving long‐term hormone replacement therapy. Gynecol Obstet Invest. 1997;44(2):124‐126. 9286727 10.1159/000291502

[12] Yonezawa T , Chen K‐H , Ghosh MK , et al. Anti‐metastatic outcome of isoform‐specific prolactin receptor targeting in breast cancer. Cancer Lett. 2015;366(1):84‐92. 26095602 10.1016/j.canlet.2015.06.010

[13] Sutherland A , Forsyth A , Cong Y , et al. The role of prolactin in bone metastasis and breast cancer cell‐mediated osteoclast differentiation. J Natl Cancer Inst. 2016;108(3):djv338. 10.1093/jnci/djv338 PMC5943829 26586670

[14] Coskun U , Gunel N , Toruner FB , et al. Serum leptin, prolactin and vascular endothelial growth factor (VEGF) levels in patients with breast cancer. Neoplasma. 2003;50(1):41‐46. 12687277

[15] Hachim IY , Hachim MY , Lopez VM , Lebrun JJ , Ali S . Prolactin receptor expression is an independent favorable prognostic marker in human breast cancer. Appl Immunohistochem Mol Morphol. 2016;24(4):238‐245. 26317306 10.1097/PAI.0000000000000178

[16] González L , Zambrano A , Lazaro‐Trueba I , et al. Activation of the unliganded estrogen receptor by prolactin in breast cancer cells. Oncogene. 2009;28(10):1298‐1308. 19169277 10.1038/onc.2008.473

[17] O'Leary KA , Jallow F , Rugowski DE , et al. Prolactin activates ERα in the absence of ligand in female mammary development and carcinogenesis in vivo. Endocrinology. 2013;154(12):4483‐4492. 24064365 10.1210/en.2013-1533 PMC3836081

[18] Ferraldeschi R , Arnedos M , Hadfield KD , et al. Polymorphisms of CYP19A1 and response to aromatase inhibitors in metastatic breast cancer patients. Breast Cancer Res Treat. 2012;133(3):1191‐1198. 22418701 10.1007/s10549-012-2010-z

[19] Koduru SV , Tiwari AK , Leberfinger A , et al. A comprehensive NGS data analysis of differentially regulated miRNAs, piRNAs, lncRNAs and sn/snoRNAs in triple negative breast cancer. J Cancer. 2017;8(4):578‐596. 28367238 10.7150/jca.17633 PMC5370502

[20] Levin ER . Extranuclear estrogen receptor's roles in physiology: lessons from mouse models. Am J Physiol Endocrinol Metab. 2014;307(2):E133‐140. 24895281 10.1152/ajpendo.00626.2013 PMC4101634

[21] Yue W , Yager JD , Wang JP , Jupe ER , Santen RJ . Estrogen receptor‐dependent and independent mechanisms of breast cancer carcinogenesis. Steroids. 2013;78(2):161‐170. 23178278 10.1016/j.steroids.2012.11.001

[22] Formisano L , Lu Y , Servetto A , et al. Aberrant FGFR signaling mediates resistance to CDK4/6 inhibitors in ER+ breast cancer. Nat Commun. 2019;10(1):1373. 30914635 10.1038/s41467-019-09068-2 PMC6435685

[23] Jiang G , Zhang S , Yazdanparast A , et al. Comprehensive comparison of molecular portraits between cell lines and tumors in breast cancer. BMC Genom. 2016;17(Suppl 7):525. 10.1186/s12864-016-2911-z PMC5001206 27556158

[24] Ojo D , Wei F , Liu Y , et al. Factors promoting tamoxifen resistance in breast cancer via stimulating breast cancer stem cell expansion. Curr Med Chem. 2015;22(19):2360‐2374. 25882671 10.2174/0929867322666150416095744

[25] Yin L , Pan X , Zhang XT , Guo YM , Wang M . Downregulation of ER‐α36 expression sensitizes HER2 overexpressing breast cancer cells to tamoxifen. Am J Cancer Res. 2015;5(2):530‐544. 25973295 PMC4396031

[26] Osborne CK , Bardou V , Hopp TA , et al. Role of the estrogen receptor coactivator AIB1 (SRC‐3) and HER‐2/neu in tamoxifen resistance in breast cancer. J Natl Cancer Inst. 2003;95(5):353‐361. 12618500 10.1093/jnci/95.5.353

[27] Shibata T , Watari K , Izumi H , et al. Breast cancer resistance to antiestrogens is enhanced by increased ER degradation and ERBB2 expression. Cancer Res. 2017;77(2):545‐556. 27879270 10.1158/0008-5472.CAN-16-1593

[28] Shen Y , Zhong J , Liu J , et al. Protein arginine N‐methyltransferase 2 reverses tamoxifen resistance in breast cancer cells through suppression of ER‐alpha36. Oncol Rep. 2018;39(6):2604‐2612. 29620287 10.3892/or.2018.6350 PMC5983932

[29] Shimoda M , Hori A , Wands JR , et al. Endocrine sensitivity of estrogen receptor‐positive breast cancer is negatively correlated with aspartate‐beta‐hydroxylase expression. Cancer Sci. 2017;108(12):2454‐2461. 28985022 10.1111/cas.13416 PMC5715250

[30] Horibata S , Rice EJ , Mukai C , et al. ER‐positive breast cancer cells are poised for RET‐mediated endocrine resistance. PLoS One. 2018;13(4):e0194023. 29608602 10.1371/journal.pone.0194023 PMC5880349

[31] Xiao T , Li W , Wang X , et al. Estrogen‐regulated feedback loop limits the efficacy of estrogen receptor‐targeted breast cancer therapy. Proc Natl Acad Sci USA. 2018;115(31):7869‐7878. 29987050 10.1073/pnas.1722617115 PMC6077722

[32] Liang Y‐K , Zeng D , Xiao Y‐S , et al. MCAM/CD146 promotes tamoxifen resistance in breast cancer cells through induction of epithelial–mesenchymal transition, decreased ERα expression and AKT activation. Cancer Lett. 2017;386:65‐76. 27838413 10.1016/j.canlet.2016.11.004

[33] Takeshita T , Yamamoto Y , Yamamoto‐Ibusuki M , et al. Prevalence of ESR1 E380Q mutation in tumor tissue and plasma from Japanese breast cancer patients. BMC Cancer. 2017;17(1):786. 29166868 10.1186/s12885-017-3779-2 PMC5700624

[34] Zhao Y , Laws MJ , Guillen VS , et al. Structurally novel antiestrogens elicit differential responses from constitutively active mutant estrogen receptors in breast cancer cells and tumors. Cancer Res. 2017;77(20):5602‐5613. 28904064 10.1158/0008-5472.CAN-17-1265 PMC5645250

[35] Jeselsohn R , Yelensky R , Buchwalter G , et al. Emergence of constitutively active estrogen receptor‐alpha mutations in pretreated advanced estrogen receptor‐positive breast cancer. Clin Cancer Res. 2014;20(7):1757‐1767. 24398047 10.1158/1078-0432.CCR-13-2332 PMC3998833

[36] Merenbakh‐Lamin K , Ben‐Baruch N , Yeheskel A , et al. D538G mutation in estrogen receptor‐alpha: A novel mechanism for acquired endocrine resistance in breast cancer. Cancer Res. 2013;73(23):6856‐6864. 24217577 10.1158/0008-5472.CAN-13-1197

[37] Wang S , Li Q , Wang Y , et al. Upregulation of circ‐UBAP2 predicts poor prognosis and promotes triple‐negative breast cancer progression through the miR‐661/MTA1 pathway. Biochem Biophys Res Commun. 2018;505(4):996‐1002. 30314706 10.1016/j.bbrc.2018.10.026

[38] Martin LA , Ribas R , Simigdala N , et al. Discovery of naturally occurring ESR1 mutations in breast cancer cell lines modelling endocrine resistance. Nat Commun. 2017;8(1):1865. 29192207 10.1038/s41467-017-01864-y PMC5709387

[39] Magnani L , Frige G , Gadaleta RM , et al. Acquired CYP19A1 amplification is an early specific mechanism of aromatase inhibitor resistance in ERalpha metastatic breast cancer. Nat Genet. 2017;49(3):444‐450. 28112739 10.1038/ng.3773 PMC5326683

[40] Cairns J , Ingle JN , Wickerham LD , Weinshilboum R , Liu M , Wang L . SNPs near the cysteine proteinase cathepsin O gene (CTSO) determine tamoxifen sensitivity in ERα‐positive breast cancer through regulation of BRCA1. PLOS Genet. 2017;13(10):e1007031. 28968398 10.1371/journal.pgen.1007031 PMC5638617

[41] Young CD , Zimmerman LJ , Hoshino D , et al. Activating PIK3CA mutations induce an epidermal growth factor receptor (EGFR)/extracellular signal‐regulated kinase (ERK) paracrine signaling axis in basal‐like breast cancer. Mol Cell Proteomics. 2015;14(7):1959‐1976. 25953087 10.1074/mcp.M115.049783 PMC4587316

[42] Li J , Lu M , Jin J , Lu X , Xu T , Jin S . miR‐449a suppresses tamoxifen resistance in human breast cancer cells by targeting ADAM22. Cell Physiol Biochem. 2018;50(1):136‐149. 30278449 10.1159/000493964

[43] Muluhngwi P , Krishna A , Vittitow SL , et al. Tamoxifen differentially regulates miR‐29b‐1 and miR‐29a expression depending on endocrine‐sensitivity in breast cancer cells. Cancer Lett. 2017;388:230‐238. 27986463 10.1016/j.canlet.2016.12.007 PMC5318263

[44] Xue X , Yang YA , Zhang A , et al. LncRNA HOTAIR enhances ER signaling and confers tamoxifen resistance in breast cancer. Oncogene. 2016;35(21):2746‐2755. 26364613 10.1038/onc.2015.340 PMC4791209

[45] Hayes EL , Lewis‐Wambi JS . Mechanisms of endocrine resistance in breast cancer: an overview of the proposed roles of noncoding RNA. Breast Cancer Res. 2015;17:40. 25849966 10.1186/s13058-015-0542-y PMC4362832

[46] Wu C , Luo J . Long non‐coding RNA (lncRNA) urothelial carcinoma‐associated 1 (UCA1) enhances tamoxifen resistance in breast cancer cells via inhibiting mTOR signaling pathway. Med Sci Monit. 2016;22:3860‐3867. 27765938 10.12659/MSM.900689 PMC5077288

[47] Peng WX , Huang JG , Yang L , Gong AH , Mo YY . Linc‐RoR promotes MAPK/ERK signaling and confers estrogen‐independent growth of breast cancer. Mol Cancer. 2017;16(1):161. 29041978 10.1186/s12943-017-0727-3 PMC5645922

[48] Simoes BM , O'Brien CS , Eyre R , et al. Anti‐estrogen resistance in human breast tumors is driven by JAG1‐NOTCH4‐dependent cancer stem cell activity. Cell Rep. 2015;12(12):1968‐1977. 26387946 10.1016/j.celrep.2015.08.050 PMC4594158

[49] Sansone P , Ceccarelli C , Berishaj M , et al. Self‐renewal of CD133(hi) cells by IL6/Notch3 signalling regulates endocrine resistance in metastatic breast cancer. Nat Commun. 2016;7:10442. 26858125 10.1038/ncomms10442 PMC4748123

[50] O'Brien CS , Howell SJ , Farnie G , Clarke RB . Resistance to endocrine therapy: are breast cancer stem cells the culprits? J Mammary Gland Biol Neoplasia. 2009;14(1):45‐54. 19252972 10.1007/s10911-009-9115-y

[51] O'Brien CS , Farnie G , Howell SJ , Clarke RB . Breast cancer stem cells and their role in resistance to endocrine therapy. Horm Cancer. 2011;2(2):91‐103. 21761332 10.1007/s12672-011-0066-6 PMC10358078

[52] Rohira AD , Yan F , Wang L , et al. Targeting SRC coactivators blocks the tumor‐initiating capacity of cancer stem‐like cells. Cancer Res. 2017;77(16):4293‐4304. 28611048 10.1158/0008-5472.CAN-16-2982 PMC5559321

[53] Jeselsohn R , Cornwell M , Pun M , et al. Embryonic transcription factor SOX9 drives breast cancer endocrine resistance. Proc Natl Acad Sci USA. 2017;114(22):E4482‐E4491. 28507152 10.1073/pnas.1620993114 PMC5465894

[54] Bergamaschi A , Madak‐Erdogan Z , Kim YJ , Choi YL , Lu H , Katzenellenbogen BS . The forkhead transcription factor FOXM1 promotes endocrine resistance and invasiveness in estrogen receptor‐positive breast cancer by expansion of stem‐like cancer cells. Breast Cancer Res. 2014;16(5):436. 25213081 10.1186/s13058-014-0436-4 PMC4303117

[55] Generali D , Berruti A , Brizzi MP , et al. Hypoxia‐inducible factor‐1alpha expression predicts a poor response to primary chemoendocrine therapy and disease‐free survival in primary human breast cancer. Clin Cancer Res. 2006;12(15):4562‐4568. 16899602 10.1158/1078-0432.CCR-05-2690

[56] Yang J , AlTahan A , Jones DT , et al. Estrogen receptor‐alpha directly regulates the hypoxia‐inducible factor 1 pathway associated with antiestrogen response in breast cancer. Proc Natl Acad Sci USA. 2015;112(49):15172‐15177. 26598706 10.1073/pnas.1422015112 PMC4679044

[57] Morotti M , Bridges E , Valli A , et al. Hypoxia‐induced switch in SNAT2/SLC38A2 regulation generates endocrine resistance in breast cancer. Proc Natl Acad Sci USA. 2019;116(25):12452‐12461. 31152137 10.1073/pnas.1818521116 PMC6589752

[58] Cheng J , Lei L , Xu J , et al. 18F‐fluoromisonidazole PET/CT: a potential tool for predicting primary endocrine therapy resistance in breast cancer. J Nucl Med. 2013;54(3):333‐340. 23401605 10.2967/jnumed.112.111963

[59] Generali D , Buffa FM , Berruti A , et al. Phosphorylated ERalpha, HIF‐1alpha, and MAPK signaling as predictors of primary endocrine treatment response and resistance in patients with breast cancer. J Clin Oncol. 2009;27(2):227‐234. 19064988 10.1200/JCO.2007.13.7083

[60] Bu L , Baba H , Yoshida N , et al. Biological heterogeneity and versatility of cancer‐associated fibroblasts in the tumor microenvironment. Oncogene. 2019;38(25):4887‐4901. 30816343 10.1038/s41388-019-0765-y

[61] Houthuijzen JM , Jonkers J . Cancer‐associated fibroblasts as key regulators of the breast cancer tumor microenvironment. Cancer Metastasis Rev. 2018;37(4):577‐597. 30465162 10.1007/s10555-018-9768-3

[62] Giussani M , Merlino G , Cappelletti V , Tagliabue E , Daidone MG . Tumor‐extracellular matrix interactions: Identification of tools associated with breast cancer progression. Semin Cancer Biol. 2015;35:3‐10. 26416466 10.1016/j.semcancer.2015.09.012

[63] Brechbuhl HM , Finlay‐Schultz J , Yamamoto TM , et al. Fibroblast subtypes regulate responsiveness of luminal breast cancer to estrogen. Clin Cancer Res. 2017;23(7):1710‐1721. 27702820 10.1158/1078-0432.CCR-15-2851 PMC5378660

[64] Diaz Bessone MI , Gattas MJ , Laporte T , Tanaka M , Simian M . The tumor microenvironment as a regulator of endocrine resistance in breast cancer. Front Endocrinol (Lausanne). 2019;10:547. 31440208 10.3389/fendo.2019.00547 PMC6694443

[65] Jansen MP , Foekens JA , van Staveren IL , et al. Molecular classification of tamoxifen‐resistant breast carcinomas by gene expression profiling. J Clin Oncol. 2005;23(4):732‐740. 15681518 10.1200/JCO.2005.05.145

[66] Pontiggia O , Sampayo R , Raffo D , et al. The tumor microenvironment modulates tamoxifen resistance in breast cancer: a role for soluble stromal factors and fibronectin through beta1 integrin. Breast Cancer Res Treat. 2012;133(2):459‐471. 21935603 10.1007/s10549-011-1766-x PMC3719875

[67] Desrochers LM , Antonyak MA , Cerione RA . Extracellular vesicles: satellites of information transfer in cancer and stem cell biology. Dev Cell. 2016;37(4):301‐309. 27219060 10.1016/j.devcel.2016.04.019 PMC4995598

[68] Sansone P , Savini C , Kurelac I , et al. Packaging and transfer of mitochondrial DNA via exosomes regulate escape from dormancy in hormonal therapy‐resistant breast cancer. Proc Natl Acad Sci USA. 2017;114(43):E9066‐E9075. 29073103 10.1073/pnas.1704862114 PMC5664494

[69] Ungefroren H , Sebens S , Seidl D , Lehnert H , Hass R . Interaction of tumor cells with the microenvironment. Cell Commun Signal. 2011;9:18. 21914164 10.1186/1478-811X-9-18 PMC3180438

[70] Choi J , Gyamfi J , Jang H , Koo JS . The role of tumor‐associated macrophage in breast cancer biology. Histol Histopathol. 2018;33(2):133‐145. 28681373 10.14670/HH-11-916

[71] Castellaro AM , Rodriguez‐Baili MC , Di Tada CE , Gil GA . Tumor‐associated macrophages induce endocrine therapy resistance in ER+ breast cancer cells. Cancers (Basel). 2019;11(2):189. 10.3390/cancers11020189 PMC6406935 30736340

[72] Joffroy CM , Buck MB , Stope MB , Popp SL , Pfizenmaier K , Knabbe C . Antiestrogens induce transforming growth factor beta‐mediated immunosuppression in breast cancer. Cancer Res. 2010;70(4):1314‐1322. 20145137 10.1158/0008-5472.CAN-09-3292

[73] Stender JD , Nwachukwu JC , Kastrati I , et al. Structural and molecular mechanisms of cytokine‐mediated endocrine resistance in human breast cancer cells. Mol Cell. 2017;65(6):1122‐1135.e1125. 28306507 10.1016/j.molcel.2017.02.008 PMC5546241

[74] Loi S , Sirtaine N , Piette F , et al. Prognostic and predictive value of tumor‐infiltrating lymphocytes in a phase III randomized adjuvant breast cancer trial in node‐positive breast cancer comparing the addition of docetaxel to doxorubicin with doxorubicin‐based chemotherapy: BIG 02–98. J Clin Oncol. 2013;31(7):860‐867. 23341518 10.1200/JCO.2011.41.0902

[75] Rugo HS , Delord JP , Im SA , et al. Safety and antitumor activity of pembrolizumab in patients with estrogen receptor‐positive/human epidermal growth factor receptor 2‐negative advanced breast cancer. Clin Cancer Res. 2018;24(12):2804‐2811. 29559561 10.1158/1078-0432.CCR-17-3452

[76] Liu L , Shen Y , Zhu X , et al. ERalpha is a negative regulator of PD‐L1 gene transcription in breast cancer. Biochem Biophys Res Commun. 2018;505(1):157‐161. 30241942 10.1016/j.bbrc.2018.09.005

[77] Anurag M , Zhu M , Huang C , et al. Immune checkpoint profiles in luminal B breast cancer (Alliance). J Natl Cancer Inst. 2020;112(7):737‐746. 31665365 10.1093/jnci/djz213 PMC7805027

[78] Bratthauer GL , Stamatakos MD , Vinh TN . Cells with minimal expression of the JAK/STAT pathway related proteins STAT5a and the prolactin receptor: evidence of an alternate prolactin receptor isoform in breast disease. Protein Pept Lett. 2010;17(1):104‐108. 20214633 10.2174/092986610789909467

[79] Gl B , Bl S . basic BRJBc, research c: reversed expression of the JAK/STAT pathway related proteins prolactin receptor and STAT5a in normal and. Abnormal Breast Epithelial Cells. 2008;1:7‐14. 10.4137/bcbcr.s549 PMC3091403 21655368

[80] Yan H , Zhao M , Huang S , et al. Prolactin inhibits BCL6 expression in breast cancer cells through a MicroRNA‐339‐5p‐dependent pathway. J Breast Cancer. 2016;19:26‐33. 27066093 10.4048/jbc.2016.19.1.26 PMC4822104

[81] Tran DD , Russell HR , Sutor SL , van Deursen J , Bram RJ . CAML is required for efficient EGF receptor recycling. Dev Cell. 2003;5(2):245‐256. 12919676 10.1016/s1534-5807(03)00207-7

[82] Guang W , Kim KC , Lillehoj EP . MUC1 mucin interacts with calcium‐modulating cyclophilin ligand. Int J Biochem Cell Biol. 2009;41(6):1354‐1360. 19135167 10.1016/j.biocel.2008.12.004 PMC3718471

[83] Lim J‐H , Kim T‐Y , Kim W‐H , Park J‐W . CAML promotes prolactin‐dependent proliferation of breast cancer cells by facilitating prolactin receptor signaling pathways. Breast Cancer Res Treat. 2011;130(1):19‐27. 21128111 10.1007/s10549-010-1274-4

[84] Knuefermann C , Lu Y , Liu B , et al. HER2/PI‐3K/Akt activation leads to a multidrug resistance in human breast adenocarcinoma cells. Oncogene. 2003;22(21):3205‐3212. 12761490 10.1038/sj.onc.1206394

[85] Yamauchi T , Kaburagi Y , Ueki K , et al. Growth hormone and prolactin stimulate tyrosine phosphorylation of insulin receptor substrate‐1, ‐2, and ‐3, their association with p85 phosphatidylinositol 3‐kinase (PI3‐kinase), and concomitantly PI3‐kinase activation via JAK2 kinase. J Biol Chem. 1998;273(25):15719‐15726. 9624169 10.1074/jbc.273.25.15719

[86] Shaw RJ , Cantley LC . Ras, PI(3)K and mTOR signalling controls tumour cell growth. Nature. 2006;441(7092):424‐430. 16724053 10.1038/nature04869

[87] Tokunaga E , Kimura Y , Mashino K , et al. Activation of PI3K/Akt signaling and hormone resistance in breast cancer. Breast Cancer. 2006;13(2):137‐144. 16755107 10.2325/jbcs.13.137

[88] Aksamitiene E , Achanta S , Kolch W , Kholodenko BN , Hoek JB , Kiyatkin A . Prolactin‐stimulated activation of ERK1/2 mitogen‐activated protein kinases is controlled by PI3‐kinase/Rac/PAK signaling pathway in breast cancer cells. Cell Signal. 2011;23(11):1794‐1805. 21726627 10.1016/j.cellsig.2011.06.014 PMC3156300

[89] Ebi H , Costa C , Faber AC , et al. PI3K regulates MEK/ERK signaling in breast cancer via the Rac‐GEF, P‐Rex1. Proc Natl Acad Sci USA. 2013;110(52):21124‐21129. 24327733 10.1073/pnas.1314124110 PMC3876254

[90] Yang Y . The Mechanism of PI3K/Akt‐PAK1 Signaling Pathway in Epidermal Growth Factor‐induced Breast Cancer Cell Migration. Department of Physiology, Nanjing Medical University; 2010.

[91] Zhu G . The Mechanism of Rac1/PAK1 Regulating Classical Wnt Signaling. Department of Biology, Tsinghua University; 2011.

[92] Hammer A , Diakonova M . Prolactin‐induced PAK1 tyrosyl phosphorylation promotes FAK dephosphorylation, breast cancer cell motility, invasion and metastasis. BMC Cell Biol. 2016;17(1):31. 27542844 10.1186/s12860-016-0109-5 PMC4992334

[93] Bilgin B , Sendur MAN , Sener Dede D , Akinci MB , Yalcin B . A current and comprehensive review of cyclin‐dependent kinase inhibitors for the treatment of metastatic breast cancer. Curr Med Res Opin. 2017;33(9):1559‐1569. 28657360 10.1080/03007995.2017.1348344

[94] Li HY , Liang JL , Kuo YL , et al. miR‐105/93‐3p promotes chemoresistance and circulating miR‐105/93‐3p acts as a diagnostic biomarker for triple negative breast cancer. Breast Cancer Res. 2017;19(1):133. 29258605 10.1186/s13058-017-0918-2 PMC5738224

[95] Arendt LM , Grafwallner‐Huseth TL , Schuler LA . Prolactin‐growth factor crosstalk reduces mammary estrogen responsiveness despite elevated ERalpha expression. Am J Pathol. 2009;174(3):1065‐1074. 19179608 10.2353/ajpath.2009.080719 PMC2665765

[96] Loibl S , Turner NC , Ro J , et al. Palbociclib combined with fulvestrant in premenopausal women with advanced breast cancer and prior progression on endocrine therapy: PALOMA‐3 results. Oncologist. 2017;22(9):1028‐1038. 28652278 10.1634/theoncologist.2017-0072 PMC5599195

[97] Cristofanilli M , DeMichele A , Giorgetti C , et al.: Predictors of prolonged benefit from palbociclib plus fulvestrant in women with endocrine‐resistant hormone receptor–positive/human epidermal growth factor receptor 2–negative metastatic breast cancer in PALOMA‐3. Eur J Cancer. 2018;104:21‐31. 30308388 10.1016/j.ejca.2018.08.011

[98] Bross PF , Cohen MH , Williams GA , Pazdur R . FDA drug approval summaries: fulvestrant. Oncologist. 2002;7(6):477‐480. 12490735 10.1634/theoncologist.7-6-477

[99] Baselga J , Im SA , Iwata H , et al. Buparlisib plus fulvestrant versus placebo plus fulvestrant in postmenopausal, hormone receptor‐positive, HER2‐negative, advanced breast cancer (BELLE‐2): a randomised, double‐blind, placebo‐controlled, phase 3 trial. Lancet Oncol. 2017;18(7):904‐916. 28576675 10.1016/S1470-2045(17)30376-5 PMC5549667

[100] Di Leo A , Johnston S , Lee KS , et al. Buparlisib plus fulvestrant in postmenopausal women with hormone‐receptor‐positive, HER2‐negative, advanced breast cancer progressing on or after mTOR inhibition (BELLE‐3): a randomised, double‐blind, placebo‐controlled, phase 3 trial. Lancet Oncol. 2018;19(1):87‐100. 29223745 10.1016/S1470-2045(17)30688-5

[101] Sabnis GJ , Goloubeva O , Chumsri S , Nguyen N , Sukumar S , Brodie AM . Functional activation of the estrogen receptor‐alpha and aromatase by the HDAC inhibitor entinostat sensitizes ER‐negative tumors to letrozole. Cancer Res. 2011;71(5):1893‐1903. 21245100 10.1158/0008-5472.CAN-10-2458 PMC3076193

[102] Yardley DA , Ismail‐Khan RR , Melichar B , et al. Randomized phase II, double‐blind, placebo‐controlled study of exemestane with or without entinostat in postmenopausal women with locally recurrent or metastatic estrogen receptor‐positive breast cancer progressing on treatment with a nonsteroidal aromatase inhibitor. J Clin Oncol. 2013;31(17):2128‐2135. 23650416 10.1200/JCO.2012.43.7251 PMC4881332

[103] Gonzalez N , Cardama GA , Comin MJ , et al. Pharmacological inhibition of Rac1‐PAK1 axis restores tamoxifen sensitivity in human resistant breast cancer cells. Cell Signal. 2017;30:154‐161. 27939839 10.1016/j.cellsig.2016.12.002

[104] Shajahan‐Haq AN , Boca SM , Jin L , et al. EGR1 regulates cellular metabolism and survival in endocrine resistant breast cancer. Oncotarget. 2017;8(57):96865‐96884. 29228577 10.18632/oncotarget.18292 PMC5722529

[105] Howell SJ , Anderson E , Hunter T , Farnie G , Clarke RB . Prolactin receptor antagonism reduces the clonogenic capacity of breast cancer cells and potentiates doxorubicin and paclitaxel cytotoxicity. Breast Cancer Res. 2008;10(4):R68. 18681966 10.1186/bcr2129 PMC2575541

[106] Agarwal N , Machiels JP , Suarez C , et al. Phase I study of the prolactin receptor antagonist LFA102 in metastatic breast and castration‐resistant prostate cancer. Oncologist. 2016;21(5):535‐536. 27091421 10.1634/theoncologist.2015-0502 PMC4861370

[107] Kelly MP , Hickey C , Makonnen S , et al. Preclinical activity of the novel anti‐prolactin receptor (PRLR) ANTIBODY‐DRUG CONJUgate REGN2878‐DM1 in PRLR‐positive breast. Cancers. 2017;16(7):1299‐1311. 10.1158/1535-7163.MCT-16-0839 28377489

